# Structures of Some Novel α-Glucosyl Diterpene Glycosides from the Glycosylation of Steviol Glycosides

**DOI:** 10.3390/molecules191220280

**Published:** 2014-12-04

**Authors:** Indra Prakash, Venkata Sai Prakash Chaturvedula

**Affiliations:** Organic Chemistry Department, The Coca-Cola Company, Global Research and Development, One Coca-Cola Plaza, Atlanta, GA 30313, USA

**Keywords:** *Stevia rebaudiana*, compositae, asteraceae, glycosylation, diterpenoid glycosides, NMR, MS, hydrolysis studies

## Abstract

Four new minor diterpene glycosides with a rare α-glucosyl linkage were isolated from a cyclodextrin glycosyltransferase glucosylated stevia extract containing more than 98% steviol glycosides. The new compounds were identified as 13-[(2-*O*-β-d-glucopyranosyl-3-*O*-(4-*O*-α-d-glucopyranosyl)-β-d-glucopyranosyl-β-d-glucopyranosyl)oxy] *ent*-kaur-16-en-19-oic acid-[(4-*O*-α-d-glucopyranosyl-β-d-glucopyranosyl) ester] (**1**), 13-[(2-*O*-β-d-glucopyranosyl-β-d-glucopyranosyl)oxy] *ent*-kaur-16-en-19-oic acid-[(4-*O*-(4-*O*-(4-*O*-α-d-glucopyranosyl)-α-d-glucopyranosyl)-α-d-glucopyranosyl)-β-d-glucopyranosyl ester] (**2**), 13-[(2-*O*-β-d-glucopyranosyl-3-*O*-(4-*O*-(4-*O*-(4-*O*-α-d-glucopyranosyl)-α-d-glucopyranosyl)-α-d-glucopyranosyl)-β-d-glucopyranosyl-β-d-glucopyranosyl)oxy] *ent*-kaur-16-en-19-oic acid β-d-glucopyranosyl ester (**3**), and 13-[(2-*O*-β-d-glucopyranosyl-3-*O*-(4-*O*-(4-*O*-(4-*O*-α-d-glucopyranosyl)-α-d-glucopyranosyl)-α-d-glucopyranosyl)-β-d-glucopyranosyl-β-d-glucopyranosyl)oxy] *ent*-kaur-16-en-19-oic acid-[(4-*O*-α-d-glucopyranosyl-β-d-glucopyranosyl) ester] (**4**) on the basis of extensive NMR and mass spectral (MS) data as well as hydrolysis studies.

## 1. Introduction

**Figure 1:**
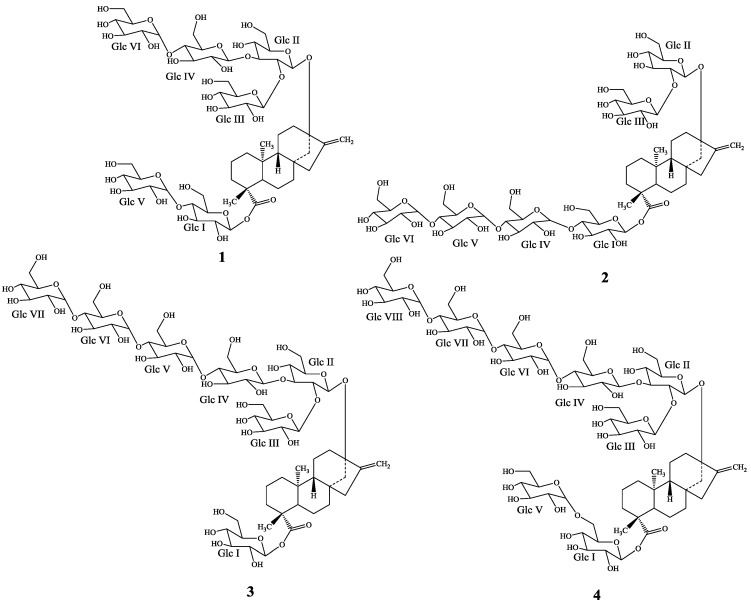
Structures of the new steviol glycosides **1**–**4**.

*Stevia rebaudiana* Bertoni (Bertoni) is a perennial shrub of the Asteraceae (Compositae) family native to certain regions of South America (Paraguay and Brazil) [1]. It is often referred to as “The sweet herb of Paraguay”, but now it is grown commercially in a number of countries, particularly in Japan, Taiwan, Korea, Thailand and Indonesia [2]. The major constituents in the leaves of *S. rebaudiana* are the potently sweet diterpenoid glycosides stevioside, rebaudiosides A and D, and dulcoside B. These compounds are all glycosides of the diterpene steviol (*ent*-13-hydroxykaur-16-en-19-oic acid) [3,4,5,6]. We have recently reported the isolation of the diterpenoid glycosides from *S. rebaudiana* having α-glucopyranosyl linkages [7]. As a part of our continuing research to discover novel natural sweeteners, we are working on the commercial extracts of the leaves of *S. rebaudiana* obtained from various suppliers across the World and we have reported several novel diterpene glycosides [8,9,10,11,12]. Purification of a glucosylated steviol glycoside mixture obtained from PureCircle (Enstek, Malaysia) resulted in the isolation of four new additional minor diterpene glycosides **1**–**4**. In this article we describe the isolation and structure elucidation of **1**–**4** based on 1D (^1^H and ^13^C) and 2D (COSY, HSQC, HSQC-TOCSY, and HMBC) NMR spectral as well as MS studies (Figure 1).

## 2. Results and Discussion

Compound **1** was isolated as a white powder and its molecular formula has been deduced as C_56_H_90_O_33_ on the basis of its positive ESI data which showed the presence of [M+H]^+^ and [M+Na]^+^ ions at *m/z* 1291.5458 and 1313.5277, respectively; this was supported by ^13^C-NMR spectral data. The ^1^H-NMR spectrum of **1** (Table 1) showed the presence of two methyl singlets at δ 1.23 and 1.26, two olefinic protons as singlets at δ 5.05 and 5.68 of an exocyclic double bond, nine methylene and two methine protons between δ 0.76–2.63 characteristic for the diterpenes belongs to the class of *ent*-kaurenes isolated earlier from the genus *Stevia* [8,9,10,11,12]. The basic skeleton of *ent-*kaurene diterpenoids was supported by the COSY and TOCSY: H-1/H-2; H-2/H-3; H-5/H-6; H-6/H-7; H-9/H-11; H-11/H-12 and HMBC: H-1/C-2, C-10; H-3/C-1, C-2, C-4, C-5, C-18, C-19; H-5/C-4, C-6, C-7, C-9, C-10, C-18, C-19, C-20; H-9/C-8, C-10, C-11, C-12, C-14, C-15; H-14/C-8, C-9, C-13, C-15, C-16 and H-17/C-13, C-15, C-16 correlations. The ^1^H-NMR spectrum of **1** also indicated the presence of six anomeric protons at δ 5.07, 5.32, 5.58, 5.81, 5.87, and 5.98 suggesting the presence of six hexose moieties in the structure. This was supported by the MS/MS spectrum of **1**, selecting the [M+H]^+^ ion at *m/z* 1,291 for fragmentation, indicated the sequential loss of six hexose moieties at *m/z* 1,129.4902, 967.4354, 805.3866, 643.3286, 481.2945, and 319.2277.

Four of the anomeric protons were well resolved at δ_H_ 5.98 (δ_C_ 95.4), 5.87 (δ_C_ 102.7), 5.81 (δ_C_ 102.9), and 5.32 (δ_C_ 104.2) in the ^1^H-NMR spectrum. One of the other two anomeric protons was observed at δ_H_ 5.07 (δ_C_ 97.8) and was partially overlapped with one of the H-17 protons, whereas the remaining anomeric proton was observed at δ_H_ 5.58 (δ_C_ 104.2) in the HSQC data but was co-suppressed with the residual H_2_O peak in the ^1^H spectrum. Two of the anomeric protons (δ_H_ 5.81 and 5.87) had small coupling constants (*J* < 4 Hz) indicating that they have an α-configuration similar to the steviol glycosides reported earlier from *S. rebaudiana* [7]; the large coupling constants observed for the other four anomeric protons appeared at δ 5.07 (d, *J* = 8.1 Hz), 5.58 (d, *J* = 7.8 Hz), 5.32 (d, *J* = 7.9 Hz), and 5.98 (d, *J* = 8.4 Hz), suggested their β-orientation as reported for steviol glycosides [8,9,10,11,12]. The anomeric proton observed at δ_H_ 5.98 showed an HMBC correlation to *C*-19 which indicated that it corresponds to the anomeric proton of Sugar-I. Similarly, the anomeric proton observed at δ_H_ 5.07 showed an HMBC correlation to *C*-13 allowing it to be assigned as the anomeric proton of Sugar-II.

**Table 1:** ^1^H-NMR chemical shift values (δ, ppm) for the compounds **1**–**4** in pyridine-*d*_5_^a−c^.

^1^H	1	2	3	4
1	0.76 t (12.1)1.74 m	0.77 t (12.5)1.74 m	0.77 t (11.5)1.76 d (12.1)	0.76 t (10.8)1.75 m
2	1.46 m2.15 m	1.45 m2.16 m	1.47 m2.18 m	1.45 d (12.3)2.17 m
3	1.04 m2.34 m	1.05 m2.34 m	1.05 m2.35 m	1.04 m2.33 d (11.7)
4	-	-	-	-
5	1.05 d (12.5)	1.06 d (12.0)	1.06 d (12.2)	1.05 d (11.4)
6	1.90 m2.33 m	1.90 m2.38 m	1.91 m2.42 m	1.91 m2.33 d (11.5)
7	1.32 m1.38 m	1.35 m	1.33 m1.37 m	1.33 m1.37 m
8	-	-	-	-
9	0.90 m	0.91 m	0.90 d (6.9)	0.90 d (6.9)
10	-	-	-	-
11	1.67 m1.70 m	1.66 m	1.68 m1.70 m	1.67 m1.70 m
12	1.91 m2.24 m	1.89 m2.24 m	1.93 m2.25 m	1.90 m2.23 m
13	-	-	-	-
14	1.79 m2.63 d (11.7)	1.79 d (11.2)2.70 d (11.1)	1.83 d (11.7)2.64 d (11.4)	1.79 d (11.0)2.63 d (11.0)
15	2.04 d (17.2)2.11 d (17.2)	2.05 d (17.3)2.11 d (17.3)	2.04 d (17.2)2.11 d (17.2)	2.03 d (17.7)2.10 d (17.7)
16	-	-	-	-
17	5.05 s5.68 s	5.10 s5.73 s	5.05 s5.68 s	5.06 s5.69 s
18	1.26 s	1.27 s	1.27 s	1.26 s
19	-	-	-	-
20	1.23 s	1.25 s	1.27 s	1.23 s
1'	5.98 d (8.4)	6.08 d (8.3)	6.07 d (8.4)	5.98 d (8.2)
2'	4.08 t (8.4)	4.13 m	4.12 m	4.08 t (8.6)
3'	4.28 m	4.40 m	4.23 m	4.28 m
4'	4.31 m	4.33 m	4.24 m	4.31 m
5'	3.74 m	3.95 m	3.98	3.72 m
6'		4.32 m	4.28 m4.43	
1''	5.07 d (8.1)	5.13 d (7.7)	5.06 d (7.6)	5.05 d (7.6)
2''	4.36 m	4.21	4.39 m	4.38 m
3''	4.30 m	4.32	4.31 m	4.36 m
4''	3.89 m	4.03	3.89 t (8.6)	3.91 t (8.5)
5''	3.77 m	3.88	3.80 t (7.6)	3.77 t (7.6)
6''	4.09 m4.30 m	4.214.31	4.10 m4.43 m	4.11 m
1'''	5.58 d (7.8)	5.32 d (7.6)	5.60 d (7.8)	5.60 d (7.8)
2'''	4.13 m	4.16	4.16 m	4.14 m
3'''	4.27 m	4.25	4.29 m	4.28 m
4'''	4.18 m	4.28	4.19 m	4.19 m
5'''	3.96 m	3.96	3.97 m	3.97 m
6'''	4.33 m4.55 m	4.394.53	4.34 m4.54 m	4.33 m4.54 m
1''''	5.32 d (7.9)	5.80 d (3.7)	5.45 d (7.8)	5.47 d (7.6)
2''''	3.97 m	4.11	4.00 m	4.00 m
3''''	4.20 m	4.57	4.32 m	4.31 m
4''''	4.14 m	4.14	4.13 m	4.13 m
5''''	3.83 m		4.03 m	4.02 m
6''''	4.30 m4.52 m		4.29 m4.54 m	4.314.54 m
1'''''	5.81 d (3.8)	5.75 d (3.8)	5.74 d (3.1)	5.87 d (3.4)
2'''''	4.16 m	4.13	4.13 m	4.14 m
3'''''	4.55 m	4.61	4.59 m	4.54 m
4'''''	4.12 m	4.21	4.15 m	4.12 m
5'''''	4.49 m	4.34	4.32 m	4.49 m
6'''''				
1''''''	5.87 d (3.7)	5.89 d (3.7)	5.78 d (3.1)	5.73 d (3.2)
2''''''	4.14 m	4.19	4.14 m	4.12 m
3''''''	4.55 m	4.57	4.62 t (9.3)	4.59 m
4''''''	4.12 m	4.14	4.21 m	4.16 m
5''''''	4.49 m	NC	4.34 m	4.32 m
6''''''	NC	NC	NC	NC
1'''''''			5.90 d (3.2)	5.78 d (3.2)
2'''''''			4.19 m	4.14 m
3'''''''			4.58 m	4.62 t (9.2)
4'''''''			4.16 m	4.21 m
5'''''''			4.54 m	4.33 m
6'''''''			NC	NC
1''''''''				5.90 d (3.2)
2''''''''				4.19 m
3''''''''				4.56 m
4''''''''				4.12 m
5''''''''				NC
6''''''''				

^a^ Assignments made on the basis of COSY, HSQC-TOCSY, HSQC and HMBC correlations; ^b^ Coupling constants are in Hz; ^c^ Chemical shift values are in δ (ppm); NC: Not Characterized.

**Table 2:** ^13^C-NMR chemical shift values (δ, ppm) for the compounds **1**–**4** in pyridine-*d*_5_^a,b^.

^13^C	1	2	3	4
1	40.7	40.8	40.8	40.8
2	19.5	19.5	19.5	19.7
3	38.4	38.4	38.4	38.4
4	44.2	44.2	44.2	44.3
5	57.3	57.3	57.4	57.4
6	22.2	22.2	22.1	22.1
7	41.6	41.7	41.8	41.7
8	-	-	-	-
9	54.1	54.0	54.1	54.1
10	39.5	39.4	39.5	39.5
11	20.5	20.7	20.6	20.5
12	37.3	36.7	37.2	37.3
13	86.7	86.6	86.9	
14	44.7	44.8	44.6	44.8
15	47.8	47.7	47.8	47.9
16	-	-	-	-
17	105.1	105.2	105.0	105.2
18	28.5	28.5	28.5	28.6
19	177.3	177.7	177.9	177.6
20	15.8	15.8	15.7	15.7
1'	95.4	95.6	95.7	95.4
2'	73.3	73.3	73.9	73.3
3'	77.9	78.0	78.7	78.0
4'	80.1	80.7	70.6	80.1
5'	77.4	ND	79.0	77.3
6'	ND	ND	ND	ND
1''	97.8	97.8	97.8	97.7
2''	80.5	83.5	80.6	80.5
3''	87.0	78.1	86.9	87.1
4''	70.1	71.8	70.2	70.1
5''	77.3	77.7	77.3	77.2
6''	62.2	62.4	ND	ND
1'''	104.2	106.1	104.3	104.2
2'''	76.1	76.5	76.1	76.0
3'''	78.1	78.0	78.2	78.0
4'''	71.8	71.5	71.8	71.8
5'''	78.4	ND	78.5	78.4
6'''	63.0	ND	ND	ND
1''''	104.2	102.7	104.2	104.1
2''''	74.5	73.5	74.5	74.6
3''''	77.8	74.7	77.6	77.8
4''''	81.3	82.0	81.9	81.9
5''''	76.8	ND	76.9	76.9
6''''	62.6	ND	ND	ND
1'''''	102.9	103.0	102.9	102.7
2'''''	74.0	73.5	73.4	73.9
3'''''	75.0	74.8	74.7	75.0
4'''''	71.6	81.4	81.9	71.7
5'''''	75.1	ND	73.3	75.1
6'''''		ND	ND	ND
1''''''	102.7	102.9	102.9	102.8
2''''''	74.0	74.1	73.2	73.4
3''''''	75.0	75.4	74.7	74.8
4''''''	71.6	71.7	81.5	81.9
5''''''	75.1	ND	73.3	73.4
6''''''		ND	ND	ND
1'''''''			103.0	102.9
2'''''''			74.0	73.4
3'''''''			74.9	74.8
4'''''''			71.8	81.5
5'''''''			75.1	73.4
6'''''''			ND	ND
1''''''''				103.0
2''''''''				74.1
3''''''''				74.9
4''''''''				71.7
5''''''''				ND
6''''''''				ND

^a^ Assignments made on the basis of COSY, HSQC-TOCSY, HSQC and HMBC correlations; ^b^ Chemical shift values are in δ (ppm); ND: Not Detected.

Acid hydrolysis of **1** afforded glucose which was identified in comparison of with standard sugars as described in the literature [13,14,15]. The ^13^C-NMR values for all the carbons were assigned on the basis of COSY, HSQC-TOCSY, HSQC and HMBC correlations and are given in Table 1 and Table 2.

**Figure 2:**
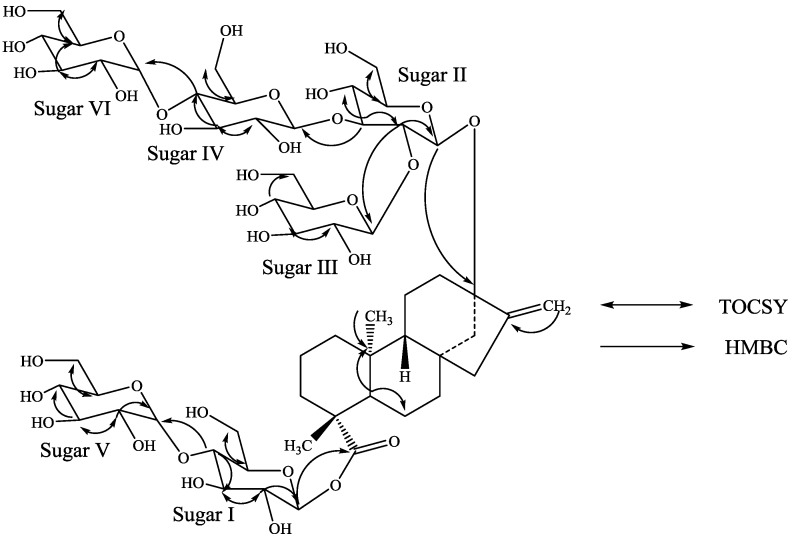
Key COSY and HMBC correlations of **1**.

Identification of sugars present in **1** and their configurations were achieved by preparing their thiocarbamoyl-thiazolidine carboxylate derivatives with l-cysteine methyl ester and *O*-tolyl isothiocyanate, and comparison of their retention times with the standard sugars as described in the literature; suggesting the sugar moieties present as d-glucopyranosyl units [16]. A close comparison of the ^1^H and ^13^C-NMR spectral data of **1** with rebaudioside A suggested that compound **1** is also a steviol glycoside which has a β-d-glucosyl substituent at C-19 and 2,3-branched β-d-glucotriosyl substituent at *C*-13 leaving the assignment of the additional two d-glucose units. The downfield chemical shift values (^1^H and ^13^C) of the C-4 position in sugars I and IV suggested the possible placement of the additional glucosyl units at these positions which was further supported by the key TOCSY and HMBC correlations as shown in Figure 2. Based on the results from chemical and spectral studies, structure of **1** was assigned as 13-[(2-*O*-β-d-glucopyranosyl-3-*O*-(4-*O*-α-d-glucopyranosyl)-β-d-glucopyranosyl-β-d-glucopyranosyl)oxy] *ent*-kaur-16-en-19-oic acid-[(4-*O*-α-d-glucopyranosyl-β-d-glucopyranosyl) ester].

The molecular formula of compound **2** was also determined to be C_56_H_90_O_33_ by high resolution mass spectral data. It’s ^1^H-NMR spectrum of **2** showed the presence of two methyl singlets, two olefinic protons as singlets of an exocyclic double bond, nine methylene and two methine protons (Table 1); similar to **1**. The negative ESI TOF MS/MS spectrum of **2**, fragmenting on the [M−H]^−^ ion at *m/z* 1,289 indicated that the most abundant ion is present at *m/z* 641.3180 and corresponds to the loss of four hexose residues likely results at *C*-19 suggested that the glycoside at *C*-19 is composed of four hexose residues and therefore the glycoside at *C*-13 should contain two hexose residues. Acid hydrolysis of **2** furnished glucose suggested the presence of six glucosyl moieties in its structure. The ^1^H-NMR spectrum of **2** also showed the anomeric protons at δ 5.13, 5.32, 5.75, 5.80, 5.89, and 6.08, identical to **1** suggesting the presence of six sugar units. The ^1^H and ^13^C-NMR values for all the carbons were assigned on the basis of COSY, HSQC and HMBC spectra and are given in Table 1 and Table 2. The large coupling constants observed for three anomeric protons of sugars I-III appeared at δ 6.08 (d, *J* = 8.3 Hz), 5.13 (d, *J* = 7.7 Hz), and 5.32 (d, *J* = 7.6 Hz) suggested their β-orientation as reported for steviol glycosides, whereas the coupling constants for the other three anomeric protons of the sugars IV, V and VI appeared at δ 5.80, 5.75, and 5.89 were obtained as 3.7, 3.8 and 3.7 Hz respectively, suggested their α-orientation, similar to **1**.

**Figure 3:**
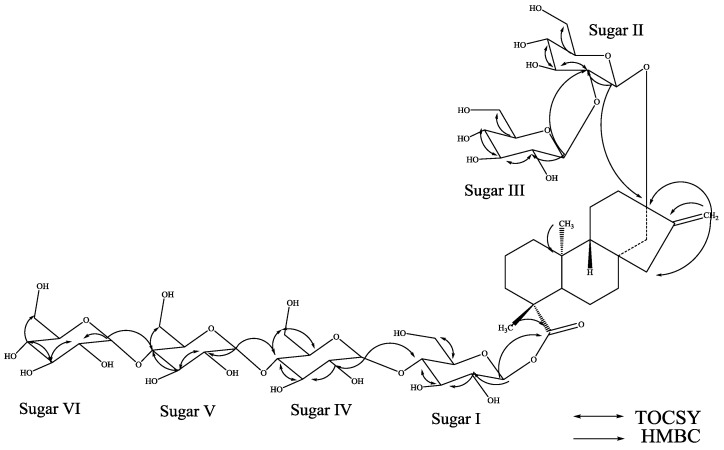
Key COSY and HMBC correlations of **2**.

Further, the identification of sugars present in **2** and their configurations were achieved by preparing their thiocarbamoyl-thiazolidine carboxylate derivatives as described for **1** suggesting the sugar moieties as d-glucopyranosylunits. From the above spectral data and hydrolysis results, it was clear that both compounds **1** and **2** are identical except for the connectivity of the glucosyl units to the basic aglycone (steviol) skeleton and the orientation. A close comparison of the ^1^H and ^13^C-NMR chemical shift values of **2** with **1**, and stevioside suggested the presence of a β-d-glucosyl substituent at *C*-19 and 2-β-d-glucobiosyl substituent at *C*-13 leaving the assignment of the additional three α-d-glucosyl units. A series of COSY, HSQC-TOCSY, HSQC and HMBC experiments suggested the placement of the other three d-glucosyl units as α-d-glucopyranosyl-(1→4)*-O-*[α-d-glucopyranosyl-(1→4)]*-O-*α-d-glucupyranosyl at the *C*-4 position of the β-d-glucosyl unit at *C*-19, as shown in Figure 3. Thus, structure of **2** was established as 13-[(2-*O*-β-d-glucopyranosyl-β-d-glucopyranosyl)oxy] *ent*-kaur-16-en-19-oic acid –[(4-*O*-(4-*O*-(4-*O*-α-d-glucopyranosyl)-α-d-glucopyranosyl)-α-d-glucopyranosyl)-β-d-glucopyranosyl ester].

The molecular formula of compound **3** was determined to be C_62_H_100_O_38_ by high resolution mass spectral data which showed [M+H]^+^ and [M+Na]^+^ ions at *m/z* 1453.6035 and 1475.5829, respectively. It’s ^1^H-NMR spectrum showed the presence of two methyl singlets, two olefinic protons as singlets of an exocyclic double bond, nine methylene and two methine protons (Table 1); similar to **1** and **2**. The ^1^H-NMR spectrum of **3** also showed the anomeric protons at δ 5.06, 5.45, 5.60, 5.74, 5.78, 5.90, and 6.07, suggesting the presence of seven sugar units. Acid hydrolysis of **3** furnished glucose suggested the presence of seven glucosyl moieties in its structure. The negative ESI TOF MS/MS spectrum of **3**, fragmenting on the [M−H]^−^ ion at *m/z* 1251 indicated that the most abundant and readily formed ion is present at *m/z* 1289.5277 corresponds to the loss of one glucose residue suggested that the glycoside at *C*-19 is composed of a single glucose residue and therefore the glycoside at *C*-13 should contain six glucose residues. The ^1^H and ^13^C-NMR values for all the carbons were assigned on the basis of COSY, HSQC and HMBC spectra and are given in Table 1 and Table 2. The large coupling constants observed for four anomeric protons of sugars I-IV appeared at δ 6.07 (d, *J* = 8.4 Hz), 5.06 (d, *J* = 7.6 Hz), 5.60 (d, *J* = 7.8 Hz), and 5.45 (d, *J* = 7.8 Hz) suggested their β-orientation as reported for steviol glycosides, whereas the coupling constants for the other three anomeric protons of the sugars V, VI and VII appeared at δ 5.74, 5.78, and 5.90 were obtained as 3.1, 3.1 and 3.2 Hz respectively, suggesting their α-orientation. Further, the identification of sugars present in **3** and their configurations were achieved by preparing their thiocarbamoyl-thiazolidine carboxylate derivatives as described for **1** suggesting the sugar moieties as d-glucopyranosyl units.

**Figure 4:**
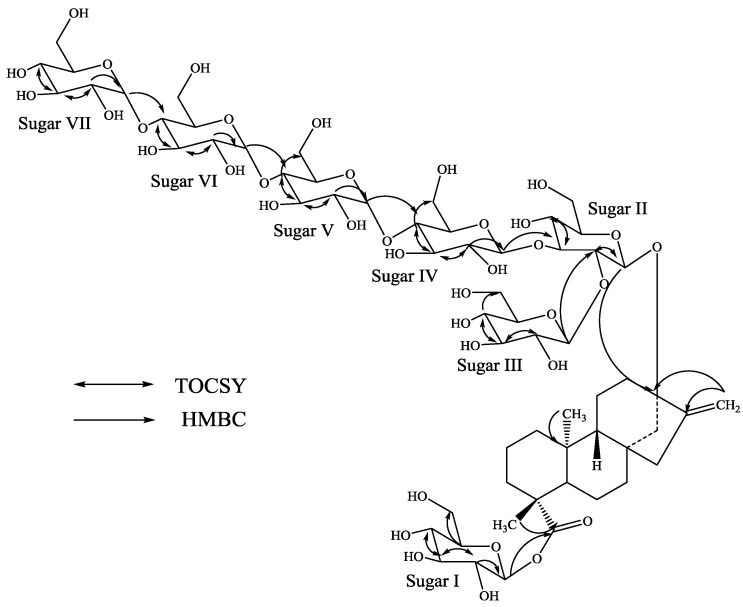
Key COSY and HMBC correlations of **3**.

A close comparison of the ^1^H and ^13^C-NMR chemical shift values of **3** with **1**, and rebaudioside A suggested the presence of a β-d-glucosyl substituent at C-19 and a 2,3-branched β-d-glucotriosyl substituent at *C*-13, leaving the assignment of the additional three α-d-glucosyl units. From the key COSY, HSQC-TOCSY, HSQC and HMBC experiments, the placement of the other three α-d-glucosyl units were assigned as α-d-glucopyranosyl-(1→4)*-O-*[α-d-glucopyranosyl-(1→4)]*-O-*α-d-glucupyranosyl to the *C*-4 position of the sugar-IV as shown in Figure 4.

The molecular formula of compound **4** was determined to be C_68_H_110_O_43 _by high resolution mass spectral data which showed [M+H]^+^ and [M+Na]^+^ ions at *m/z* 1615.6498 and 1637.6302, respectively; this was supported by ^13^C-NMR spectral data. It’s ^1^H-NMR spectrum showed the presence of two methyl singlets, two olefinic protons as singlets of an exocyclic double bond, nine methylene and two methine protons (Table 1); similar to **1**–**3**. The ^1^H-NMR spectrum of **4** showed the anomeric protons at δ 5.05, 5.47, 5.60, 5.73, 5.78, 5.87, 5.90, and 5.98, suggesting the presence of eight sugar units. Acid hydrolysis of **4** furnished glucose suggested the presence of eight glucosyl moieties in its structure. The negative ESI TOF MS/MS spectrum of **4**, fragmenting on the [M−H]^−^ ion at *m/z* 1613 indicated that the most abundant and readily formed ion is present at *m/z* 1289.5223 which corresponds to the loss of two glucose residues likely results at *C*-19 suggested that the structure of **4** is composed of two glucose residues at *C*-19 and therefore *C*-13 must contain six glucose residues. The ^1^H and ^13^C-NMR values for all the carbons were assigned on the basis of COSY, HSQC and HMBC spectra and are given in Table 1 and Table 2. The large coupling constants observed for the four anomeric protons of sugars I-IV appeared at δ 5.98 (d, *J* = 8.2 Hz), 5.05 (d, *J* = 7.6 Hz), 5.60 (d, *J* = 7.8 Hz), and 5.47 (d, *J* = 7.6 Hz) suggested their β-orientation as reported for steviol glycosides,whereas the coupling constants for the other four anomeric protons of the sugars V, VI, VII and VIII appeared at δ 5.87, 5.73, 5.78, and 5.90 were obtained as 3.4, 3.2, 3.2 and 3.2 Hz respectively, suggested their α-orientation.

**Figure 5:**
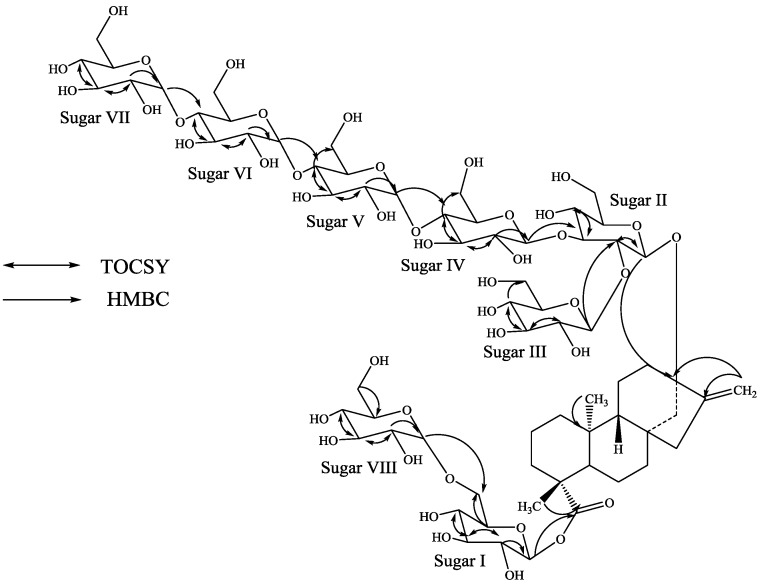
Key COSY and HMBC correlations of **4**.

Further, the identification of sugars present in **4** and their configurations were achieved by preparing their thiocarbamoyl-thiazolidine carboxylate derivatives as described for **1** suggesting the sugar moieties as d-glucopyranosyl units. A close comparison of the ^1^H and ^13^C-NMR chemical shift values of **4** with **1**–**3**, and rebaudioside A suggested the presence of a β-d-glucosyl substituent at *C*-19 and a 2,3-branched β-d-glucotriosyl substituent at *C*-13, leaving the assignment of the additional four α-d-glucosyl units. From the key COSY, HSQC-TOCSY, HSQC and HMBC experiments, the placement of the other four α-d-glucosyl units were assigned as α-d-glucopyranosyl-(1→4)*-O-*[α-d-glucopyranosyl-(1→4)]*-O-*α-d-glucupyranosyl to the *C*-4 position of the sugar-IV with an additional α-d-glucupyranosyl attached to the *C*-6 position of the sugar-I as shown in Figure 5.

## 3. Experimental Section

### 3.1. General

NMR spectra were acquired on a Bruker Avance DRX 500 MHz instrument with a 5 mm inverse detection probe using standard pulse sequences. The NMR spectrum was referenced to the residual solvent signal (δ_H_ 8.71, δ_C_ 149.9 for pyridine-*d*_5_), chemical shifts are given in δ (ppm), and coupling constants are reported in Hz. MS and MS/MS data were generated with a Waters Premier Quadrupole Time-of-Flight (Q-Tof) mass spectrometer equipped with an electrospray ionization source operated in the positive-ion mode and ThermoFisher Discovery OrbiTrap in the positive mode electrospray. Samples were diluted with water: acetonitrile (1:1) containing 0.1% formic acid and introduced via infusion using the onboard syringe pump. Analytical HPLC was carried out with a Waters 600E multisolvent delivery system using Luna C_18_ column (250 × 10 mm, 5 μm) and Phenomenex Luna C_18_ (150 × 4.6 mm, 5 μm) column. The details of various columns and other parameters used for HPLC purification for methods 1–3 (Table 3) are given below:

Column: Phenomenex Prodigy ODS(3) with a Phenomenex guard column, 250 × 21.2 mm, 5 μm (p/n 00G-4097-P0); UV Detection: 210 nm; Mobile Phase A: H_2_O; Mobile Phase B: Acetonitrile; Flow Rate: 20 mL/min; Injection volume: 1500 µL at 40 mg/mL; Detection was by UV (210 nm).

Column: Phenomenex spherex diol, 250 × 10 mm, 5 µm (p/n 00G-0021-NO); Column Temp: 25 °C; Mobile Phase A: H_2_O; Mobile Phase B: Acetonitrile; Flow Rate: 5.0 mL/min; Injection volume: 150 µL prepared in H_2_O; Detection was by UV (210 nm).

Column: Atlantis C_18_ with guard column, 250 × 10 mm, 5 µm (p/n 186003694); Column Temp: 25 °C; Mobile Phase A: H_2_O; Mobile Phase B: Acetonitrile; Flow Rate: 5.0 mL/min; Injection volume: 150 µL prepared in H_2_O; Detection was by UV (210 nm).

### 3.2. Plant Material

A sample of glucosylated steviol glycosides was obtained from PureCircle Ltd. (Bandar Enstek, Negreri Sembilan, Malaysia) which was prepared by the cyclodextrin glycosyltransferase (produced by *Bacillus stearothermophilus*) of the stevia extract with content of stevioside, rebaudioside A, rebaudioside C, and dulcoside more than 98% [17].

### 3.3. Isolation

An aliquot of glucosylated steviol glycosides (2 g) was taken to isolate new compounds **1**–**4**. A preliminary round of purification was performed using HPLC Method 1 (Table 3) and the material eluting at 16.84, 17.47, and 19.45 min were collected, which on evaporation under vacuum furnished Fractions 1–3 respectively. A second round of purification of Fraction 1 was performed using a diol column with method 3 and the peak eluted at 14.17 min was collected from multiple injections, pooled, and dried by rotary evaporation under reduced pressure which on final purification using HPLC Method 2 over several injections with an atlantis C_18_ column and dried by rotary evaporation under reduced pressure to provide **4** (*t_R_* 9.87 min, 3.2 mg). Purification of Fraction 2 using HPLC method 3 with a diol column and collected the peaks eluted at 10.36 and 12.01 min which on concentration followed by a final round of purification using HPLC method 2 with an atlantis C_18_ column over multiple runs furnished **2** (*t_R_* 13.14 min, 4.2 mg), and **3** (*t_R_* 13.33 min, 3.8 mg) respectively. Similarly, final purification of Fraction 3 using HPLC method 2 over multiple injections with an atlantis C_18_ column and the pure compound was dried by rotary evaporation under reduced pressure to provide **1** (*t_R_* 18.52 min, 3.6 mg).

**Table 3:** RP-HPLC methods for the isolation and purification of steviol glycosides **1**–**4**.

HPLC Method	Time (min)	% of Mobile Phase A	% of Mobile Phase B
Method 1	0.0	75	25
	8.5	75	25
	10.0	71	29
	16.5	70	30
	18.5	66	34
	24.5	66	34
	25.0	0	100
	30.0	0	100
Method 2	0.0	72	28
	60.0	72	28
Method 3	0.0	20	80
	100.0	20	80

*13-[(2-O-β-d-Glucopyranosyl-3-O-(4-O-α-d-glucopyranosyl)-β-d-glucopyranosyl-β-d-glucopyranosyl)oxy]* *ent-kaur-16-en-19-oic acid-[(4-O-α-d-glucopyranosyl-β-d-glucopyranosyl) ester]* (**1**). White powder; ^1^H-NMR (500 MHz, pyridine-*d_5_*, δ ppm) and ^13^C-NMR (125 MHz, pyridine-*d_5_*, δ ppm) spectroscopic data see Table 1 and Table 2; HRMS (M+H)^+^ *m/z* 1291.5458 (calcd. for C_56_H_91_O_33_: 1291.5443); (M+Na)^+^ *m/z* 1313.5277 (calcd. for C_56_H_9_O_33_Na: 1313.5262).

*13-[(2-O-β-d-Glucopyranosyl-β-d-glucopyranosyl)oxy] ent-kaur-16-en-19-oic acid-[(4-O-(4-O-(4-O-α-d-glucopyranosyl)-α-d-glucopyranosyl)-α-d-glucopyranosyl)-β-d-glucopyranosyl ester]* (**2**). White powder; ^1^H-NMR (500 MHz, pyridine-*d_5_*, δ ppm) and ^13^C-NMR (125 MHz, pyridine-*d_5_*, δ ppm) spectroscopic data see Table 1 and Table 2; HRMS (M+H)^+^ *m/z* 1291.5479 (calcd. for C_56_H_91_O_33_: 1291.5443); (M+Na)^+^ *m/z* 1313.5286 (calcd. for C_56_H_9_O_33_Na: 1313.5262).

*13-[(2-O-β-d-Glucopyranosyl-3-O-(4-O-(4-O-(4-O-α-d-glucopyranosyl)-α-D-glucopyranosyl)-α-d-glucopyranosyl)-β-d-glucopyranosyl-β-d-glucopyranosyl)oxy] ent-kaur-16-en-19-oic acid β-d-glucopyranosyl ester* (**3**). White powder; ^1^H-NMR (500 MHz, pyridine-*d_5_*, δ ppm) and ^13^C-NMR (125 MHz, pyridine-*d_5_*, δ ppm) spectroscopic data see Table 1 and Table 2; HRMS (M+H)^+^ *m/z* 1453.6035 (calcd. for C_62_H_101_O_38_: 1453.5971); (M+Na)^+^ *m/z* 1475.5829 (calcd. for C_62_H_100_O_38_Na: 1475.5790).

*13-[(2-O-β-d-glucopyranosyl-3-O-(4-O-(4-O-(4-O-α-d-glucopyranosyl)-α-d-glucopyranosyl)-α-d-glucopyranosyl)-β-d-glucopyranosyl-β-d-glucopyranosyl)oxy] ent-kaur-16-en-19-oic acid-[(4-O-α-d-glucopyranosyl-β-d-glucopyranosyl) ester]* (**4**). White powder; ^1^H-NMR (500 MHz, pyridine-*d_5_*, δ ppm) and ^13^C-NMR (125 MHz, pyridine-*d_5_*, δ ppm) spectroscopic data see Table 1 and Table 2; HRMS (M+H)^+^ *m/z* 1615.6498 (calcd. for C_68_H_111_O_43_: 1615.6499); (M+Na)^+^ *m/z* 1637.6302 (calcd. for C_68_H_110_O_43_Na: 1637.6319).

*Acid Hydrolysis of Compounds* **1**–**4**. To a solution of each compound **1**–**4** (250 μg) in MeOH (3 mL) was added 3 mL of 5% H_2_SO_4_ and the mixture was refluxed for 8 hours. The reaction mixture was then neutralized with saturated sodium carbonate and extracted with ethyl acetate (EtOAc) (2 × 15 mL) to give an aqueous fraction containing sugars and an EtOAc fraction containing the aglycone part. The aqueous phase was concentrated and compared with standard sugars using the TLC systems EtOAc/*n*-butanol/water (2:7:1) and CH_2_Cl_2_/MeOH/water (10:6:1); the sugar was identified as d-glucose in all four experiments [13,14,15]. 

#### *General Procedure for Acid Hydrolysis and Determination of Sugar Configuration in* **1**–**4**

Each compound **1**–**4** (500 μg) was hydrolyzed with 0.5 M HCl (0.5 mL) for 1.5 h. After cooling, the mixture was passed through an Amberlite IRA400 column and the eluate was lyophilized. The residue was dissolved in pyridine (0.25 mL) and heated with l-cysteine methyl ester HCl (2.5 mg) at 60 °C for 1.5 h. Then, *O*-tolyl isothiocyanate (12.5 µL) was added to the mixture and heated at 60 °C for an additional 1.5 h. The reaction mixture was analyzed by HPLC: column Phenomenex Luna C_18_, 150 × 4.6 mm (5 µm); 25% acetonitrile-0.2% TFA water, 1 mL/min; UV detection at 250 nm. The sugar was identified as d-glucose (*t*_R_, 12.26, 12.43, 12.46 and 12.51 min) in all compounds **1**–**4** [authentic samples, d-glucose (*t*_R_, 12.32) and L-glucose (*t*R, 11.08 min)] [16].

## 4. Conclusions

Four new minor diterpenoid steviol glycosides were isolated from a glucosylated steviol glycoside mixture obtained by cyclodextrin glycosyltransferase treatment of a stevia extract obtained from PureCircle. The new compounds were identified as 13-[(2-*O*-β-d-glucopyranosyl-3-*O*-(4-*O*-α-d-glucopyranosyl)-β-d-glucopyranosyl-β-d-glucopyranosyl)oxy] *ent*-kaur-16-en-19-oic acid-[(4-*O*-α-d-glucopyranosyl-β-d-glucopyranosyl) ester] (**1**), 13-[(2-*O*-β-d-glucopyranosyl-β-d-glucopyranosyl)oxy] *ent*-kaur-16-en-19-oic acid-[(4-*O*-(4-*O*-(4-*O*-α-d-glucopyranosyl)-α-d-glucopyranosyl)-α-d-glucopyranosyl)-β-d-glucopyranosyl ester] (**2**), 13-[(2-*O*-β-d-glucopyranosyl-3-*O*-(4-*O*-(4-*O*-(4-*O*-α-d-glucopyranosyl)-α-d-glucopyranosyl)-α-d-glucopyranosyl)-β-d-glucopyranosyl-β-d-glucopyranosyl)oxy] *ent*-kaur-16-en-19-oic acid β-d-glucopyranosyl ester (**3**), and 13-[(2-*O*-β-d-glucopyranosyl-3-*O*-(4-*O*-(4-*O*-(4-*O*-α-d-glucopyranosyl)-α-d-glucopyranosyl)-α-d-glucopyranosyl)-β-d-glucopyranosyl-β-d-glucopyranosyl)oxy] *ent*-kaur-16-en-19-oic acid-[(4-*O*-α-d-glucopyranosyl-β-d-glucopyranosyl) ester] (**4**), respectively, on the basis of extensive NMR and mass spectroscopic data and chemical studies. To the best of our knowledge this is the first report of the isolation of these four new steviol glycosides **1**–**4**.

## Supplementary Materials

Supplementary materials can be accessed at: http://www.mdpi.com/1420-3049/19/12/20280/s1.
