# Supplementary material for: Structures of Some Novel α-Glucosyl Diterpene Glycosides from the Glycosylation of Steviol Glycosides
Source: Molecules. 2014 Dec 4;19(12):20280–94. doi: 10.3390/molecules191220280 (PMC6271715; doi:10.3390/molecules191220280)

Supplementary Materials

Figure S1. 1D and 2D NMR spectra of 13-[(2-*O*-β-D-glucopyranosyl-3-*O*-(4-*O*-α-D-glucopyranosyl)-β-D-glucopyranosyl-β-D-glucopyranosyl)oxy] *ent*-kaur-16-en-19-oic acid-[(4-*O*-α-D-glucopyranosyl-β-D-glucopyranosyl) ester] (**1**).

(**a**) ^{1}H-NMR spectrum of **1**.

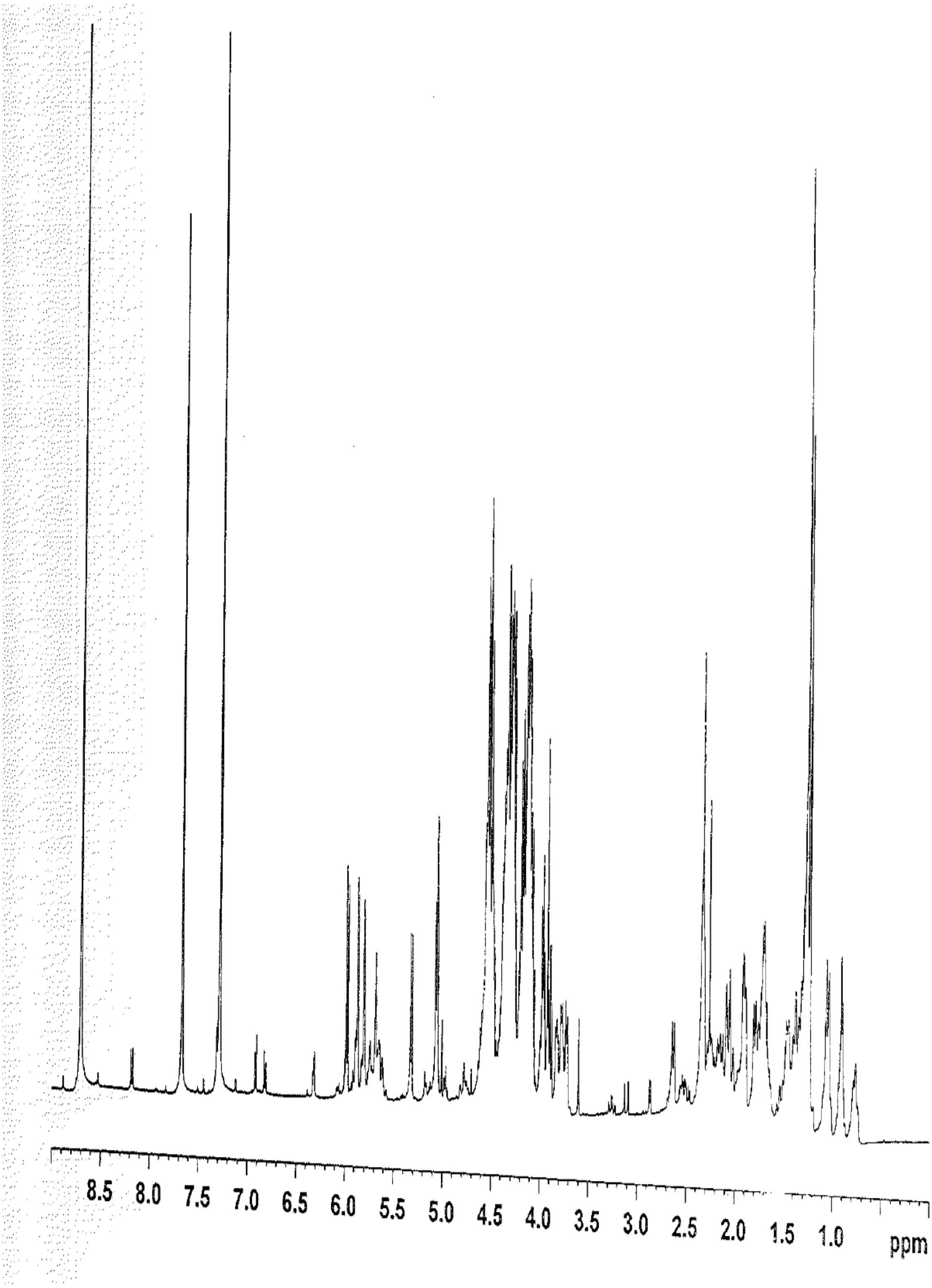

(**b**) ^{1}H-^{1}H COSY spectrum of **1**.

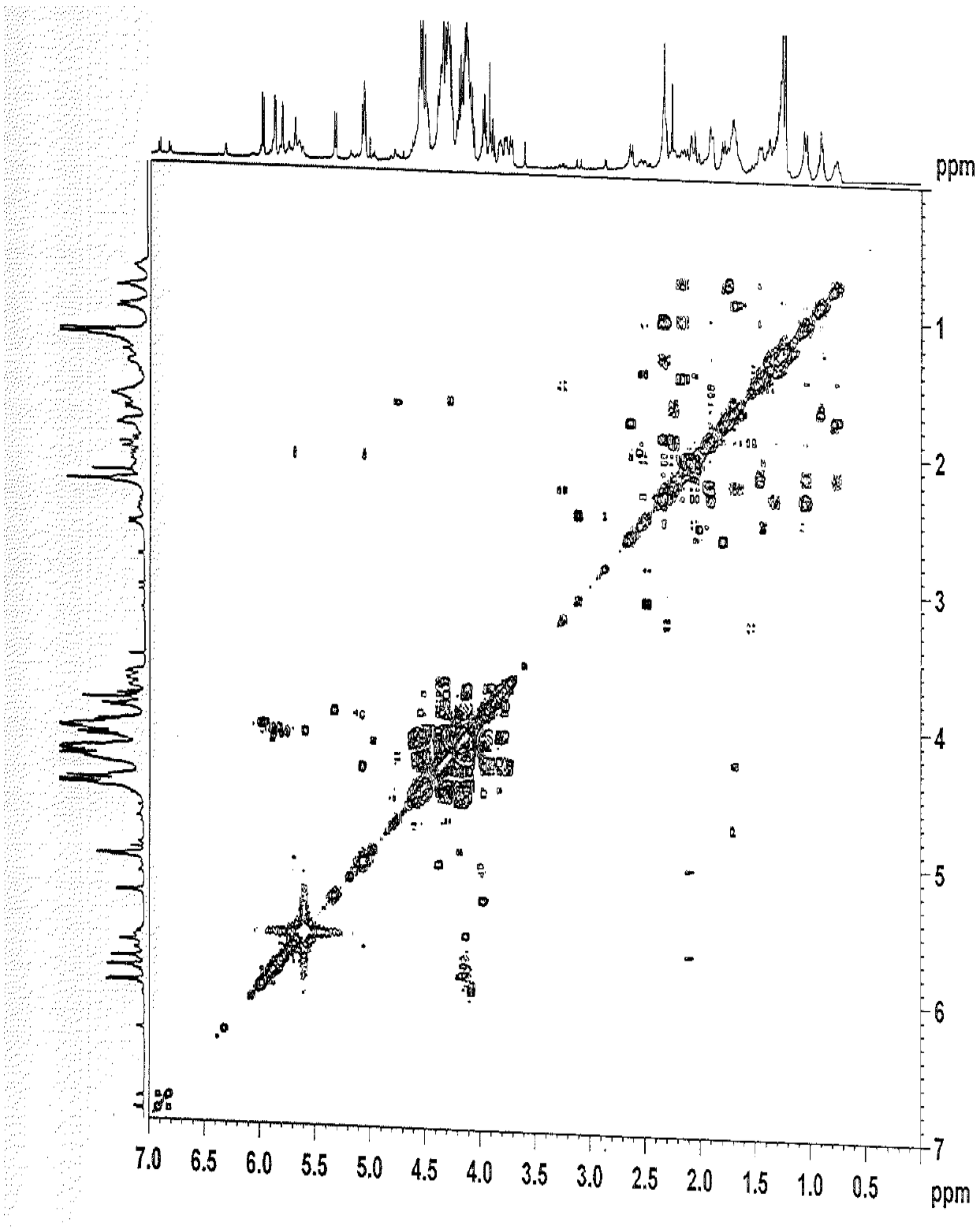

(**c**) HSQC-TOCSY spectrum of **1**.

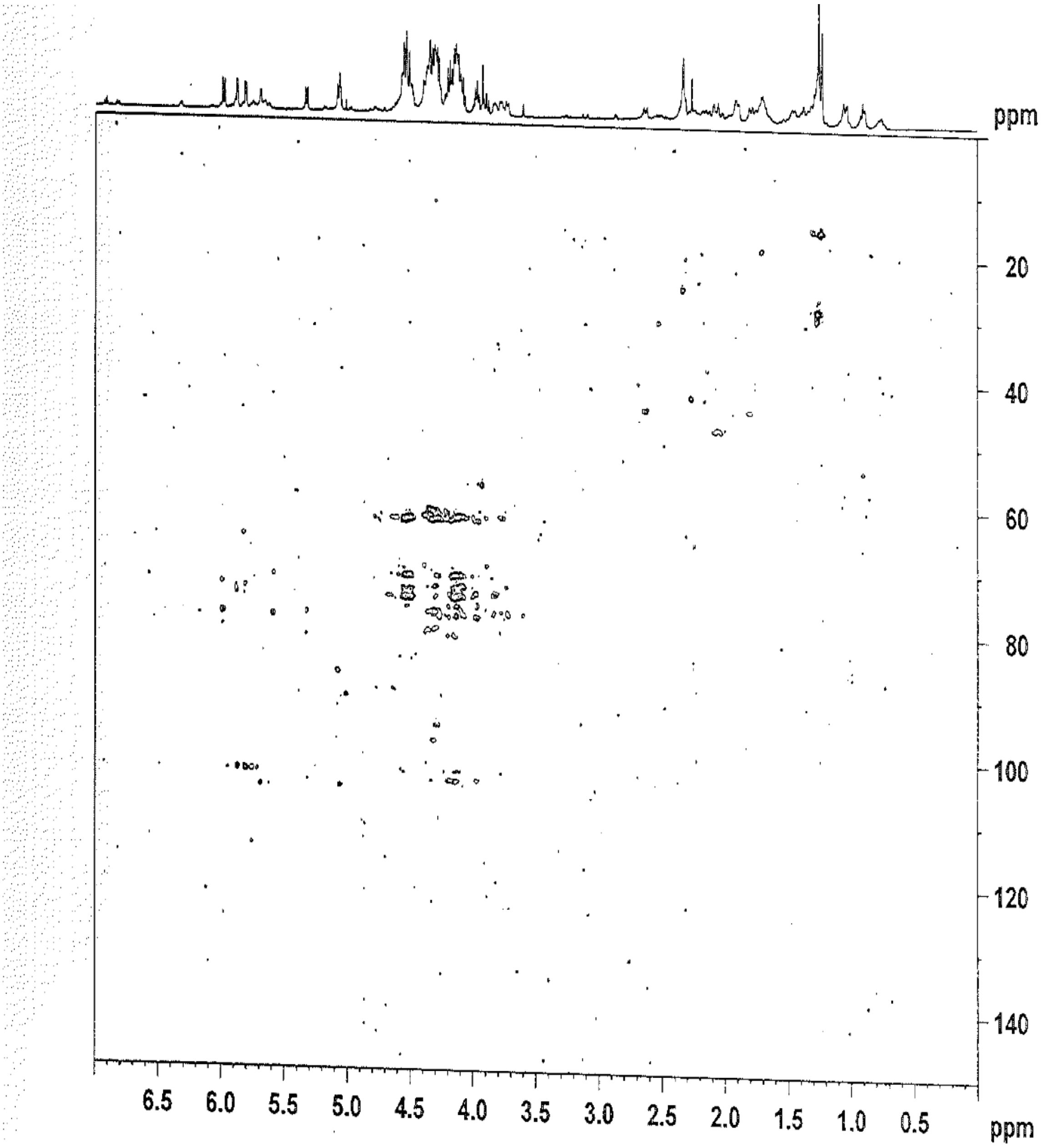

(**d**) ^{1}H-^{13}C HSQC spectrum of **1**.

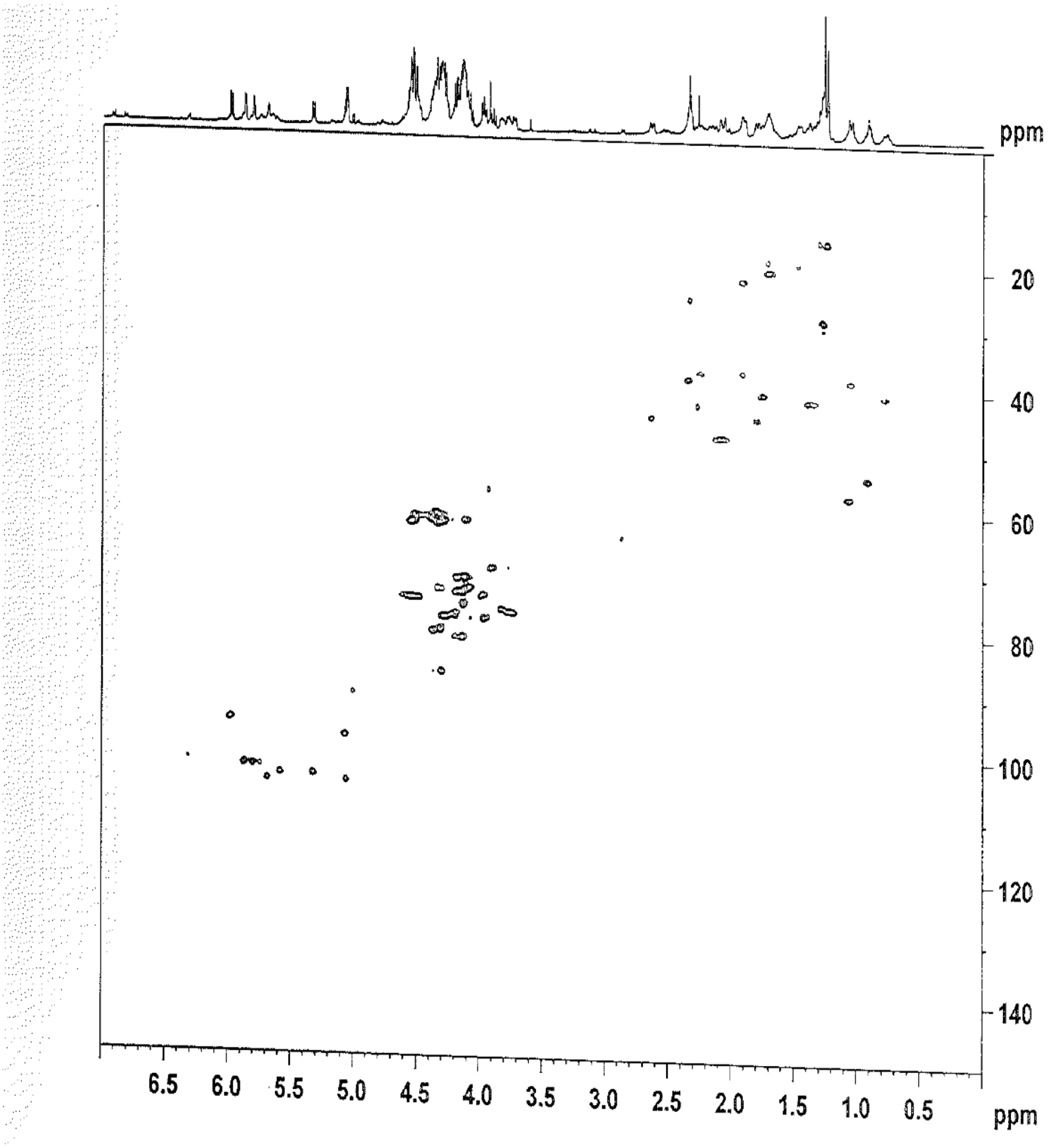

(**e**) ^{1}H-^{13}C HMBC spectrum of **1**.

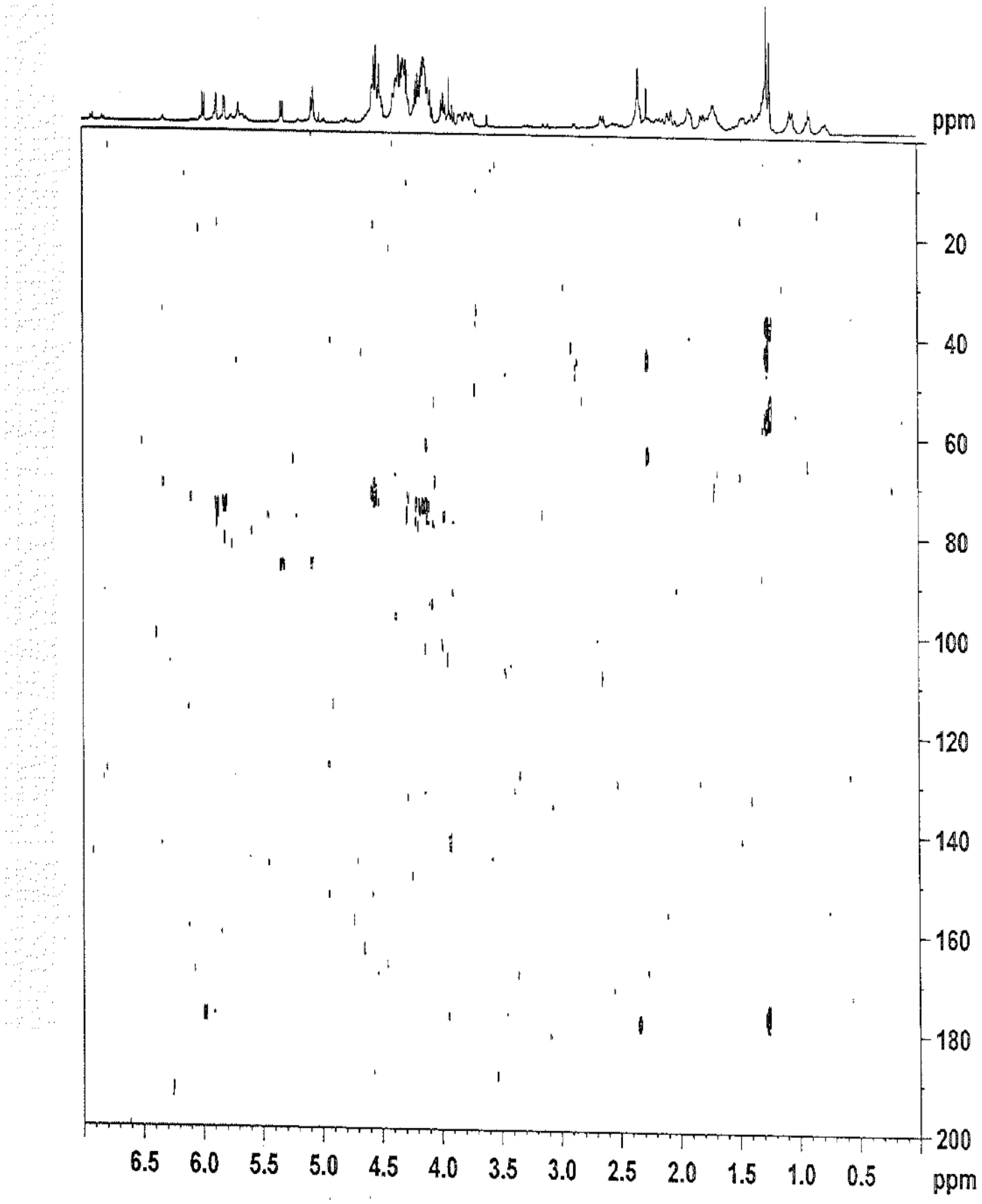

(**f**) ^{1}H-^{13}C HMBC spectrum of **1** (Expansion Version).

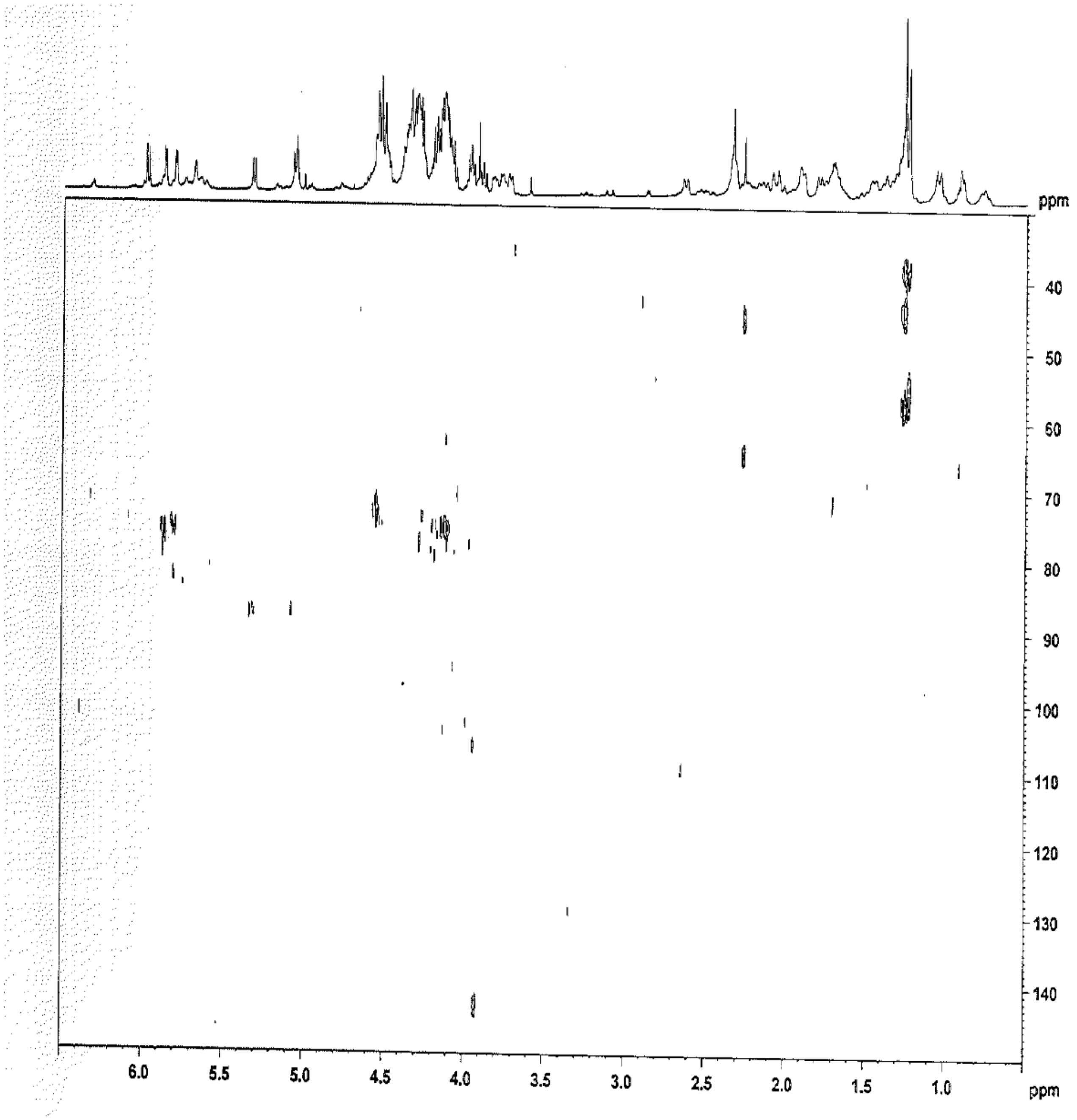

Figure S2. 1D and 2D NMR spectra of 13-[(2-*O*-β-D-glucopyranosyl-β-D-glucopyranosyl)oxy] *ent*-kaur-16-en-19-oic acid-[(4-*O*-(4-*O*-(4-*O*-α-D-glucopyranosyl)-α-D-glucopyranosyl)-α-D-glucopyranosyl)-β-D-glucopyranosyl ester] (**2**).

(**a**) ^{1}H-NMR spectrum of **2**.

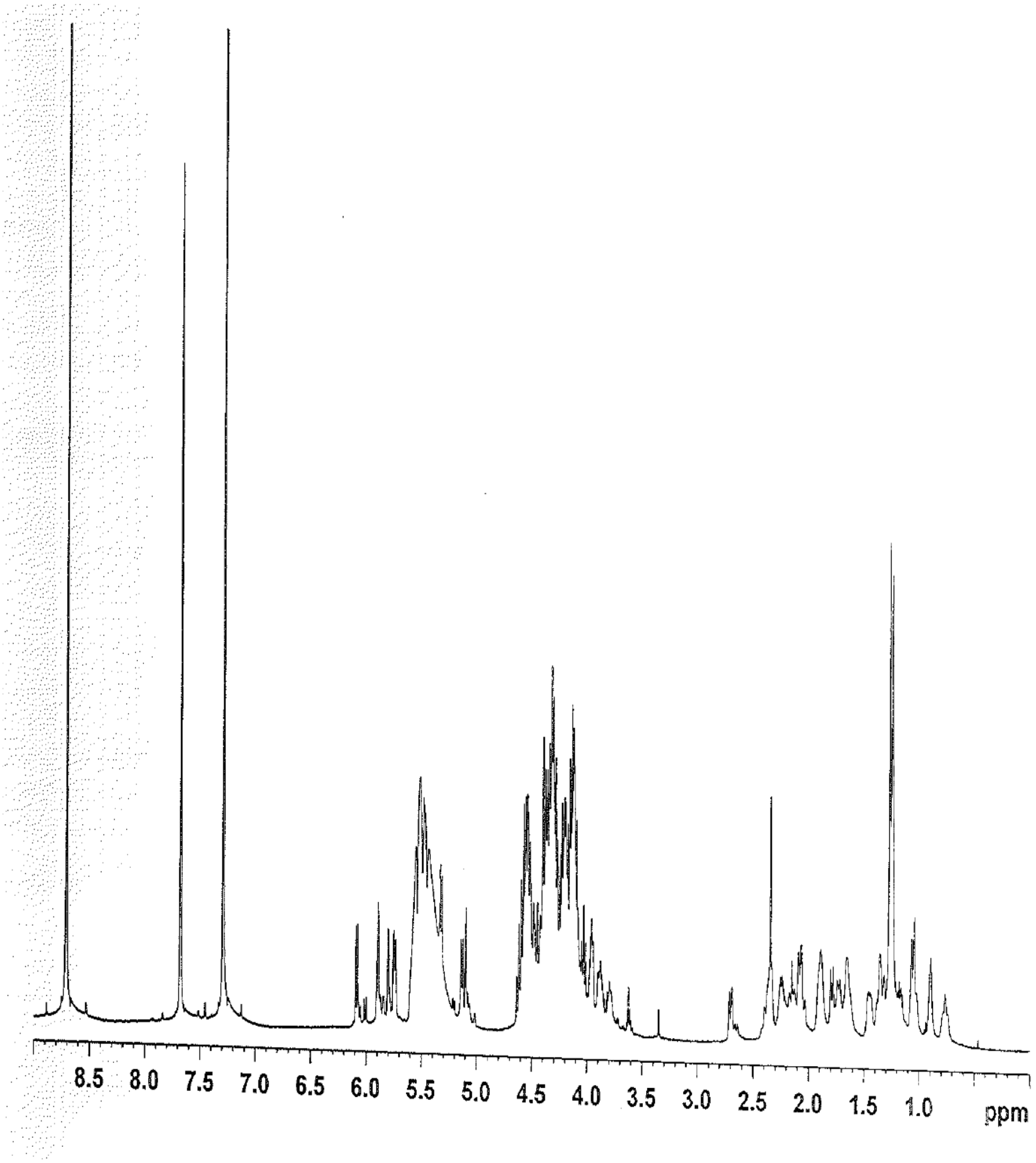

(**b**) ^{1}H-^{1}H COSY spectrum of **2**.

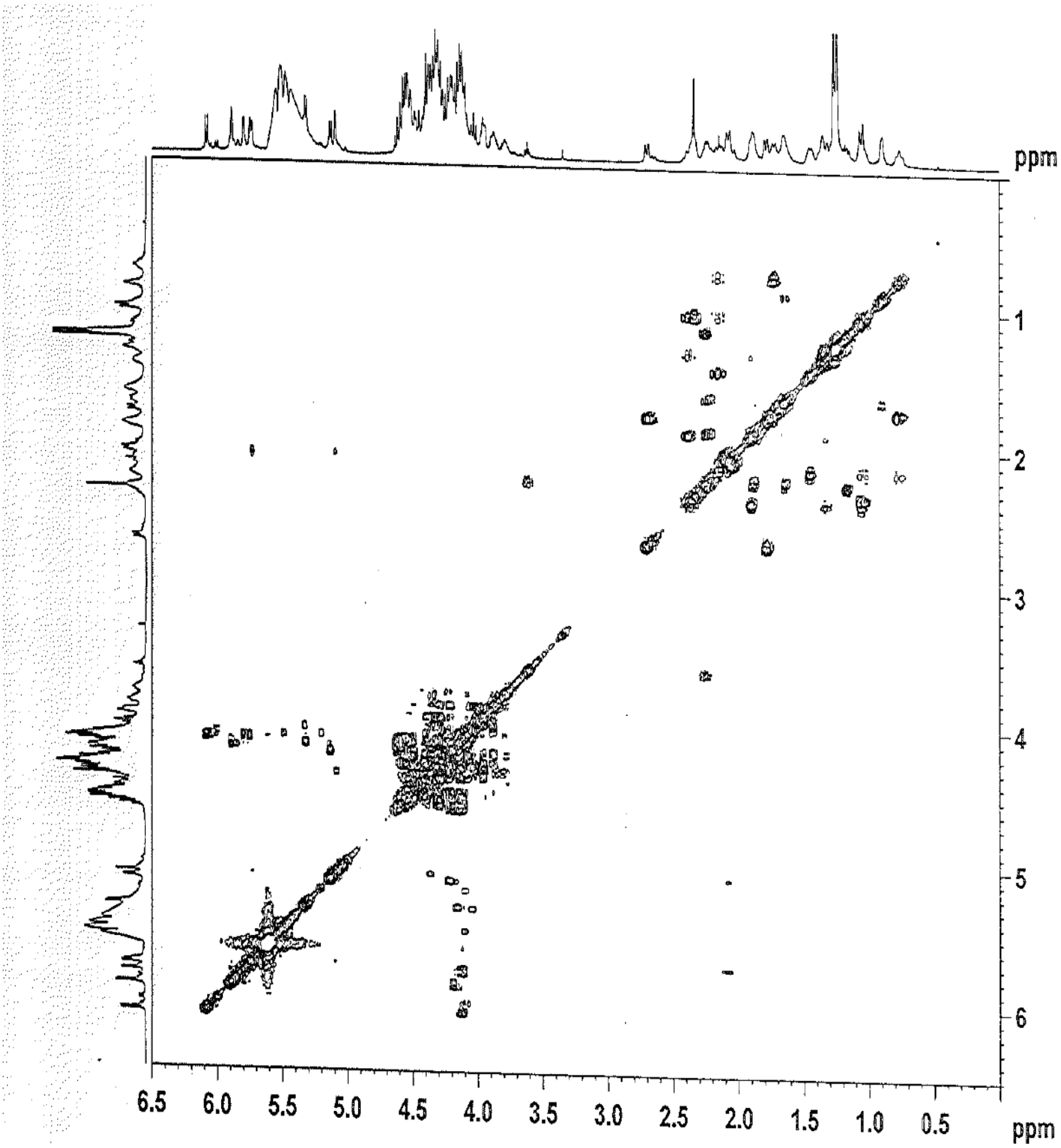

(**c**) HSQC-TOCSY spectrum of **2**.

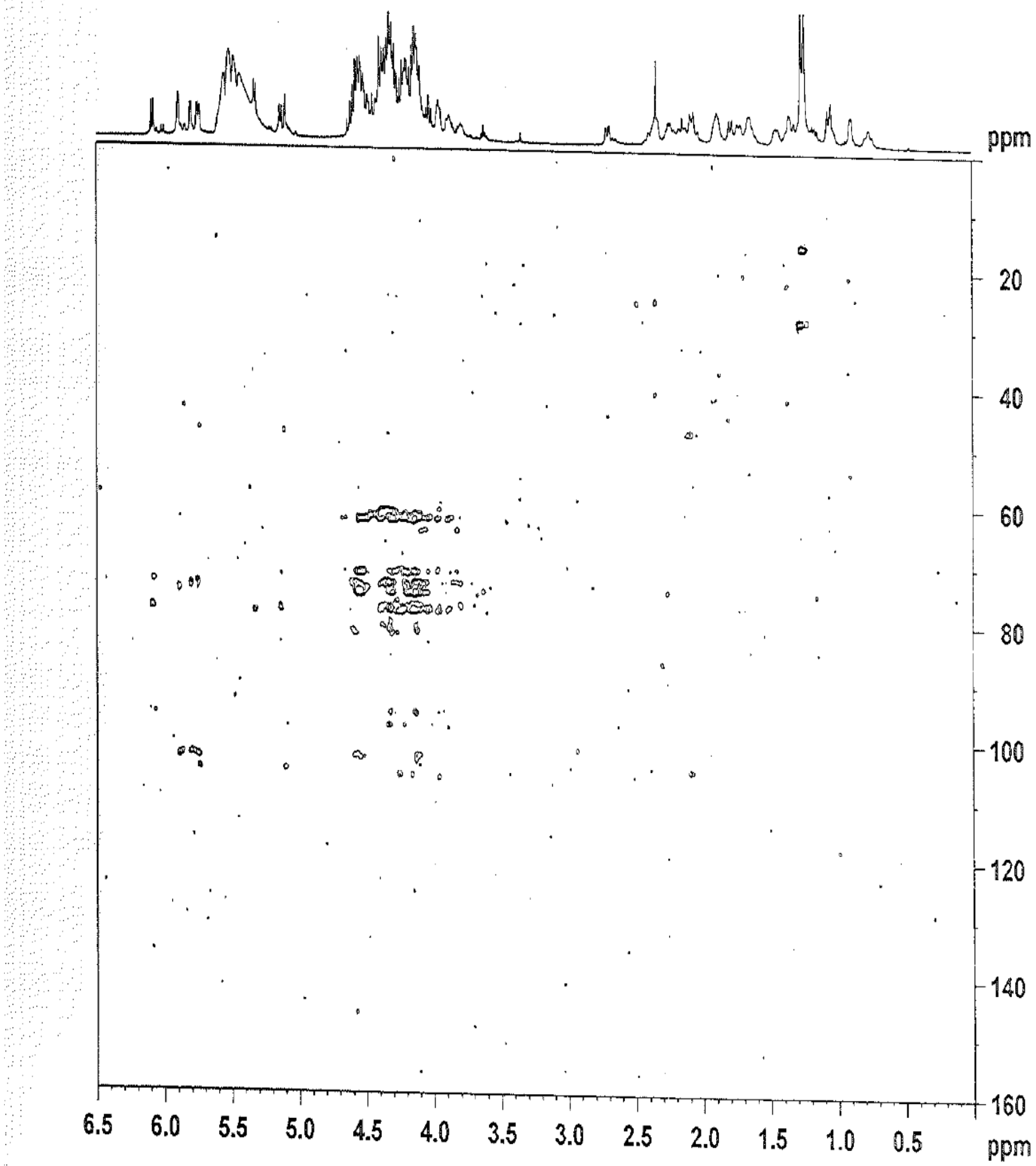

(**d**) HSQC-TOCSY spectrum of **2** (Zoom Version).

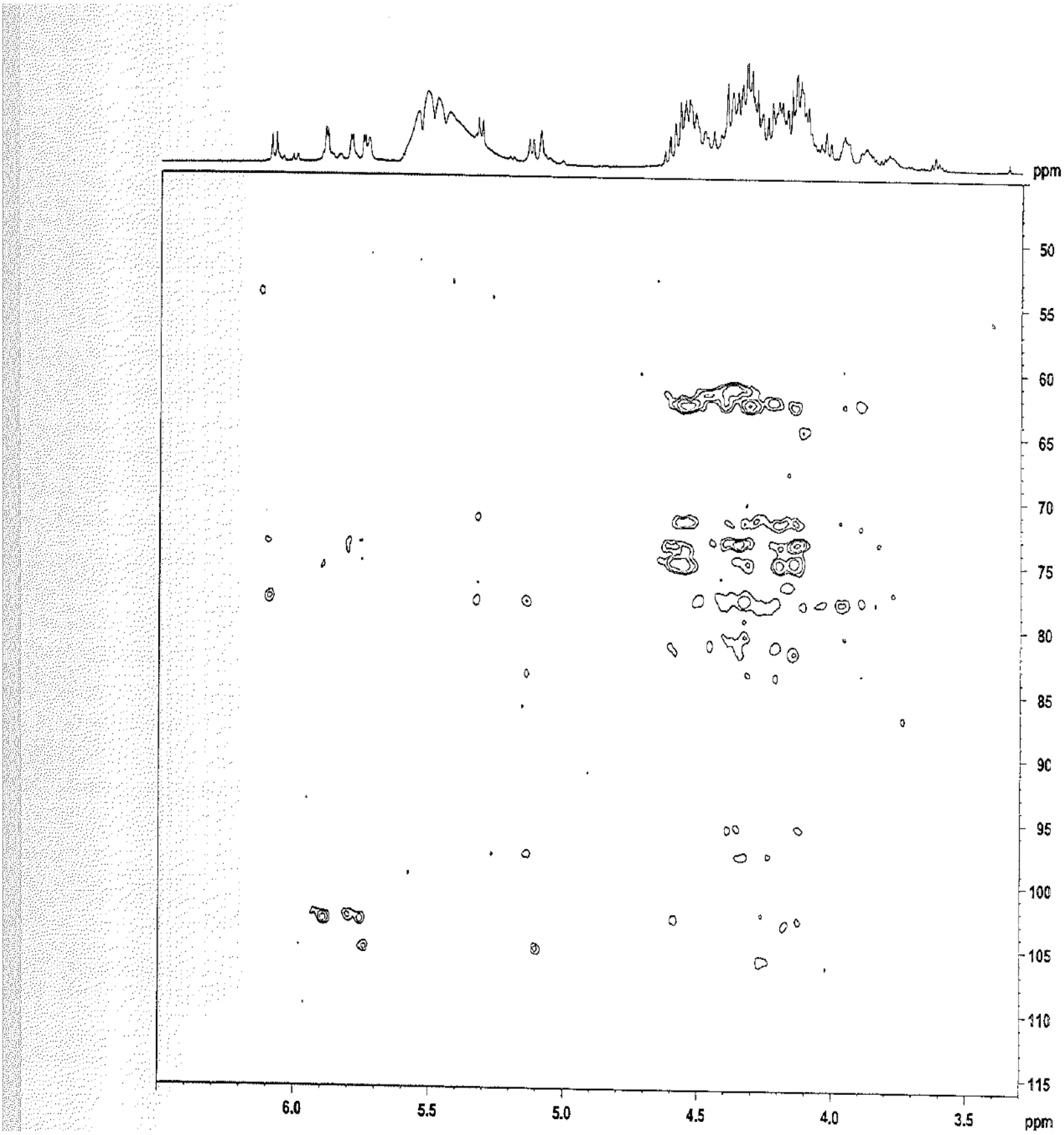

(**e**) ^{1}H-^{13}C HSQC spectrum of **2**.

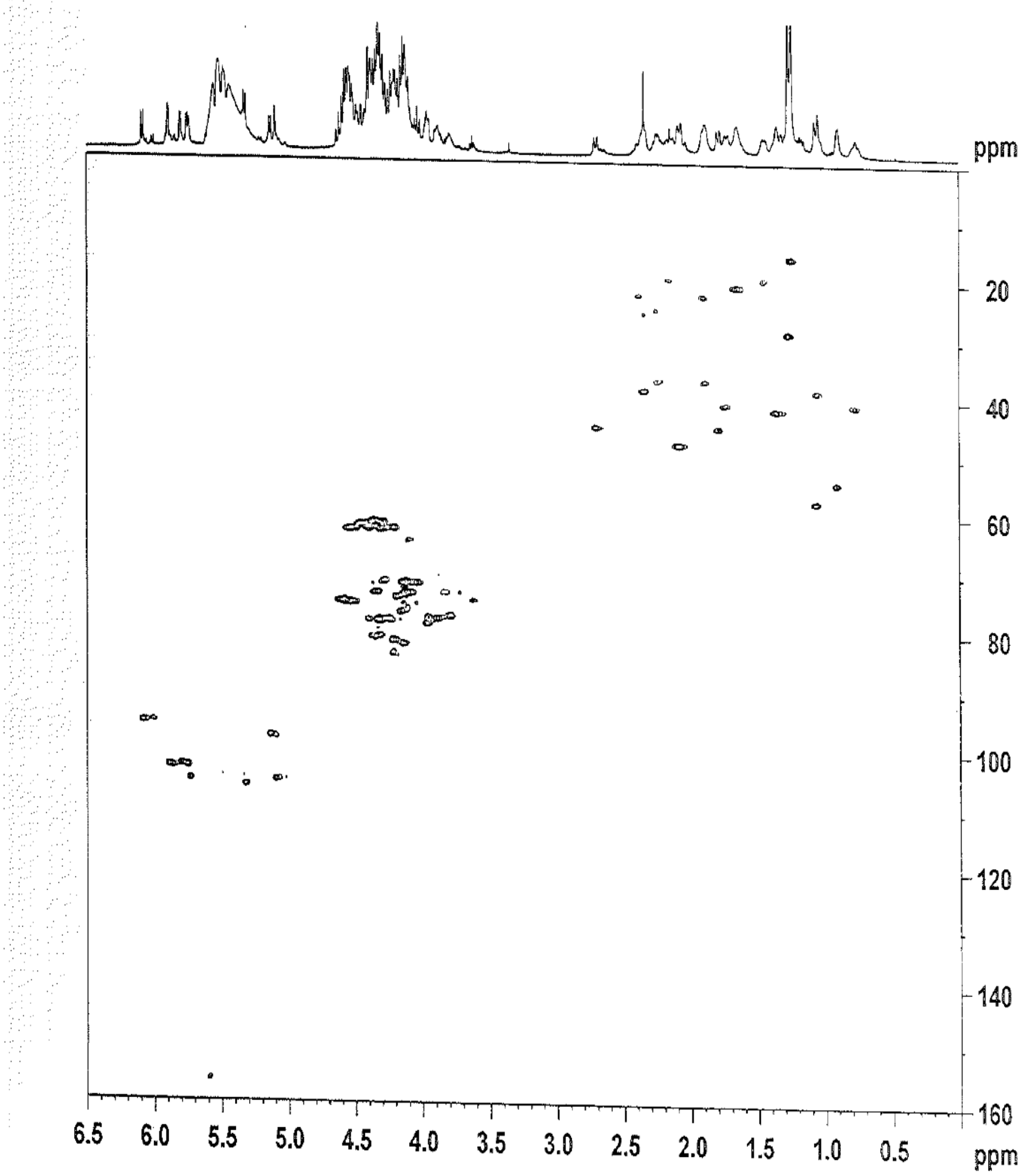

(**f**) ^{1}H-^{13}C HSQC spectrum of **2** (Zoom Version).

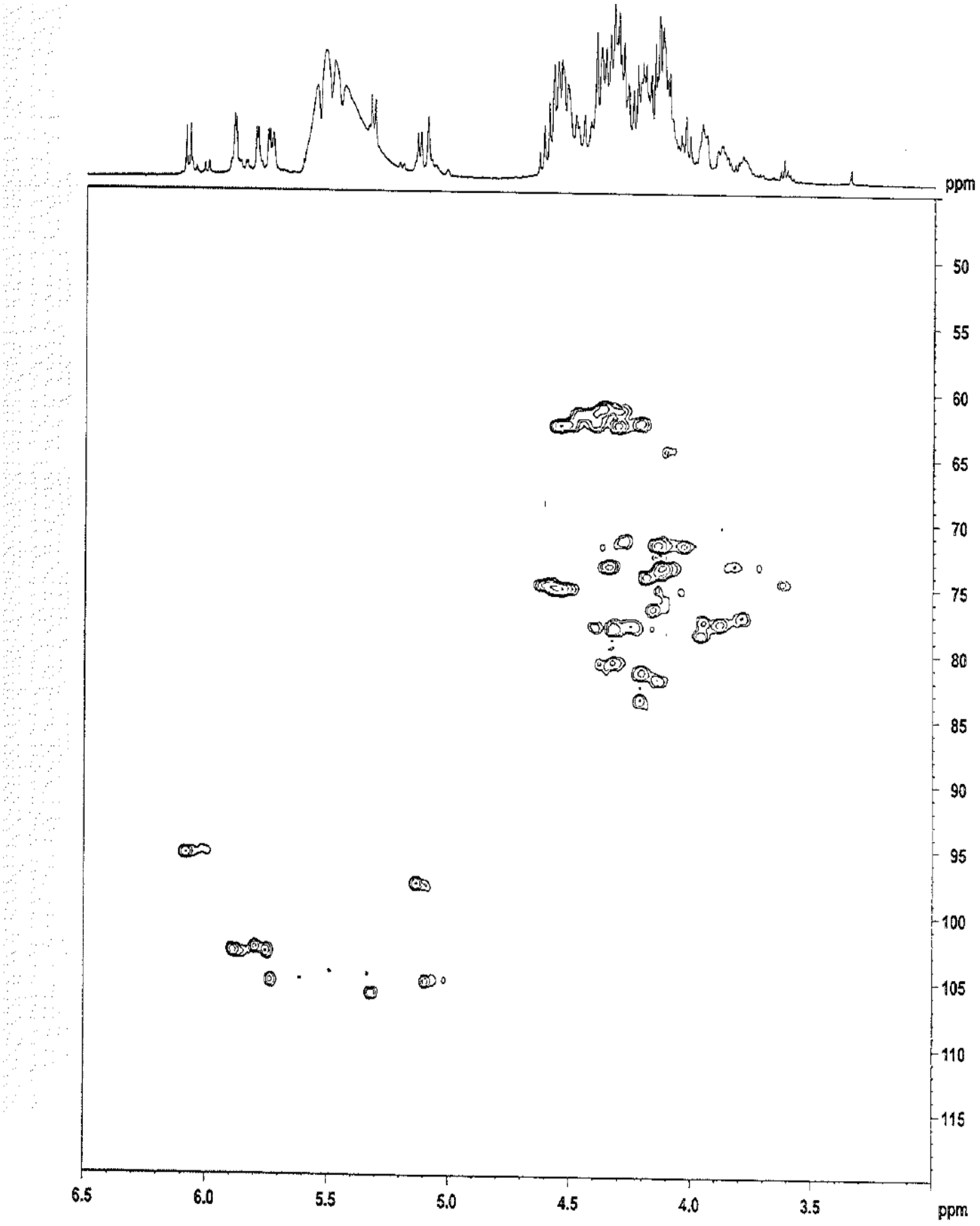

(g) ^{1}H-^{13}C HMBC spectrum of **2**.

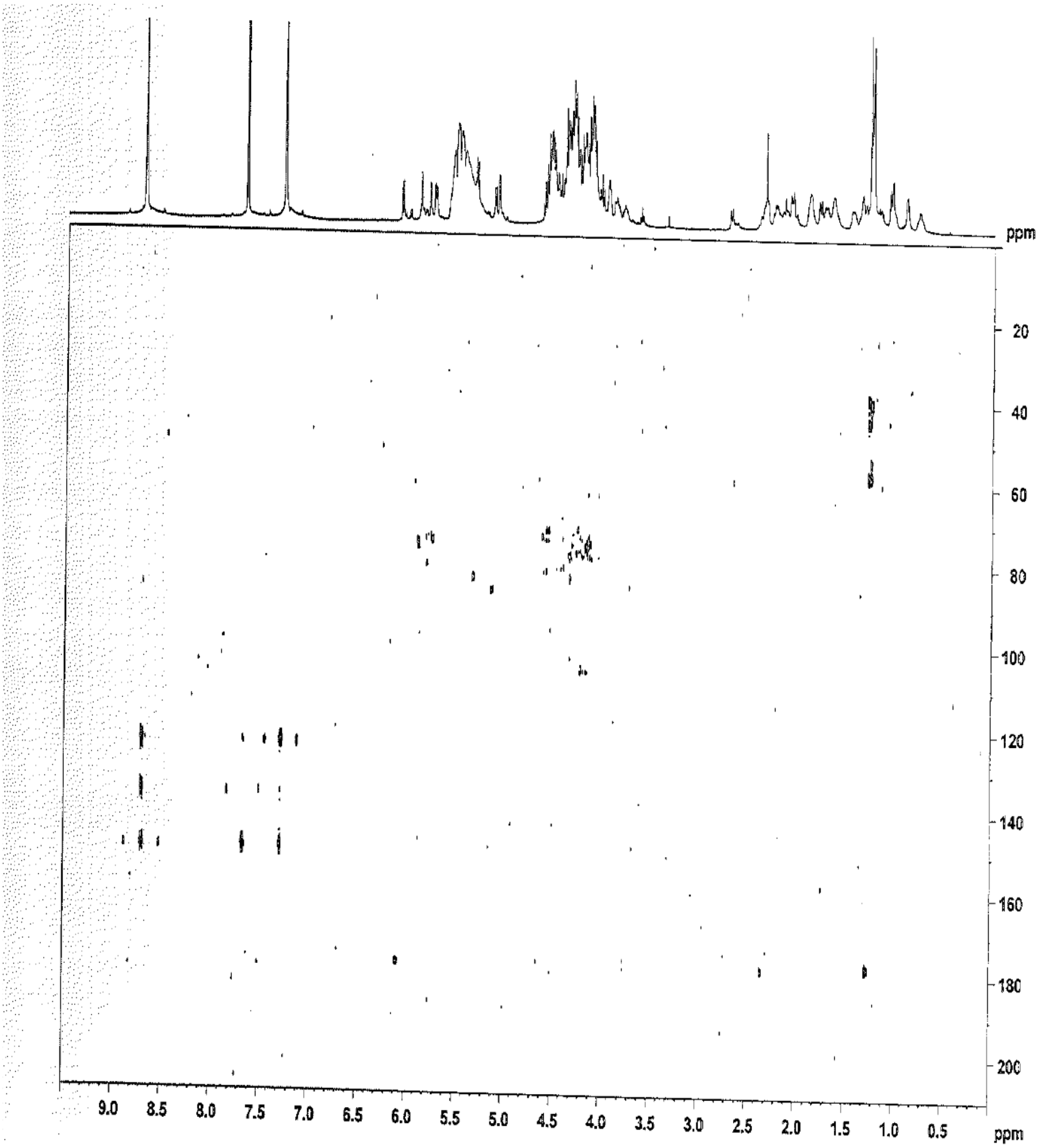

(**h**) ^{1}H-^{13}C HMBC spectrum of **2** (Zoom Version).

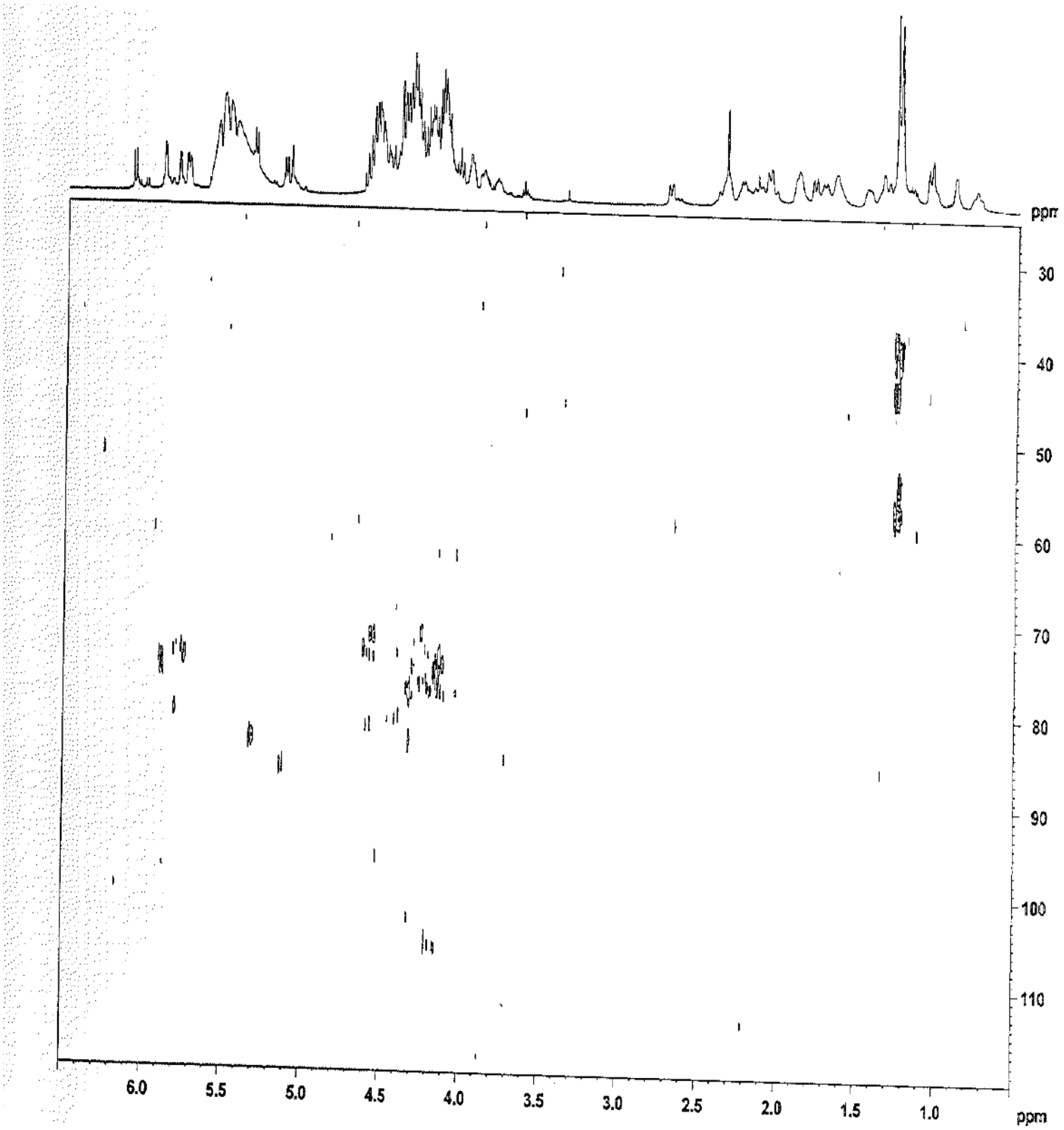

Figure S3. 1D and 2D NMR spectra of 13-[(2-*O*-β-D-glucopyranosyl-3-*O*-(4-*O*-(4-*O*-(4-*O*-α-D-glucopyranosyl)-α-D-glucopyranosyl)-α-D-glucopyranosyl)-β-D-glucopyranosyl-β-D-glucopyranosyl) oxy] *ent*-kaur-16-en-19-oic acid β-D-glucopyranosyl ester (**3**).

(**a**) ^{1}H-NMR spectrum of **3**.

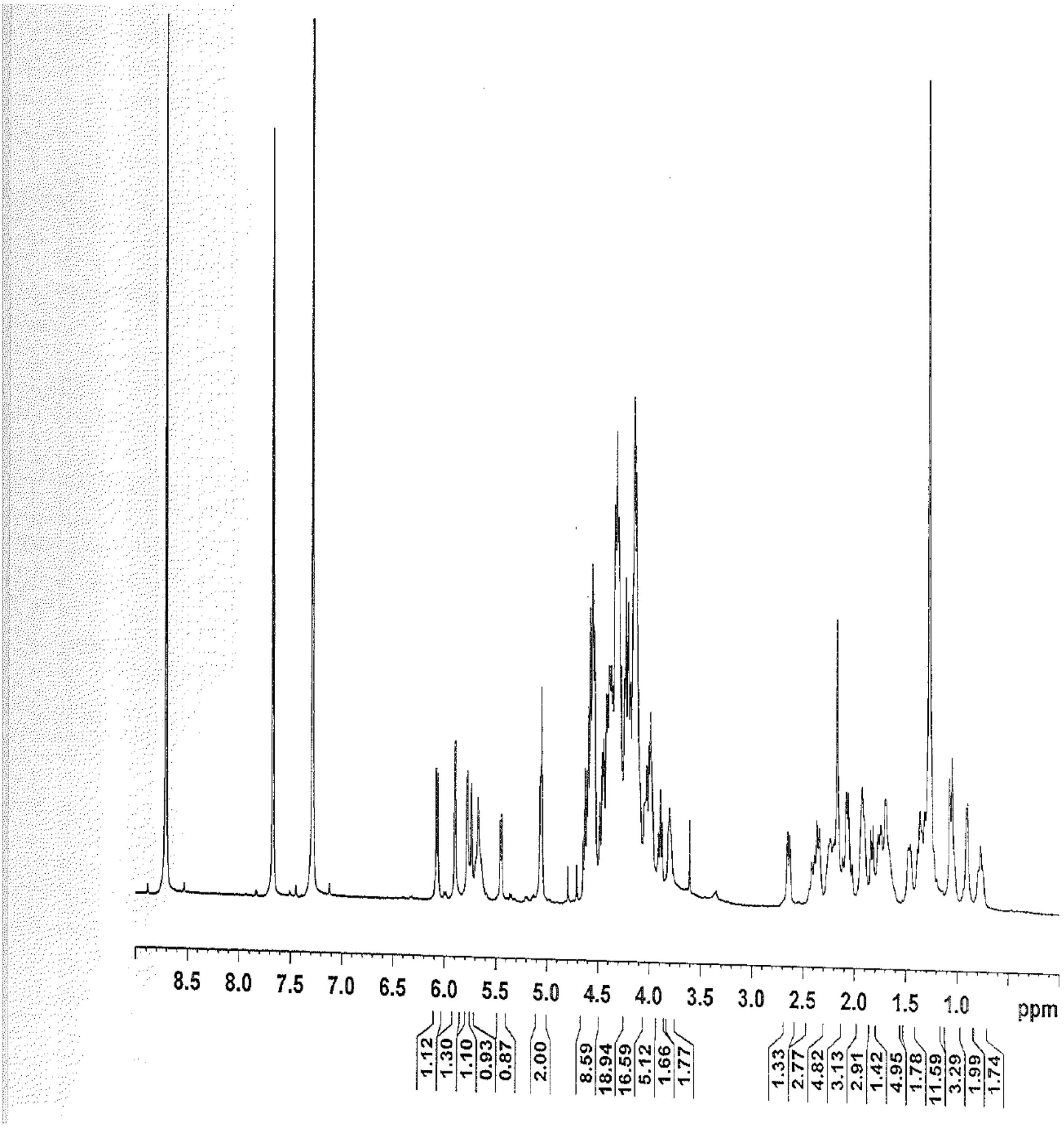

(**b**) ^{1}H-^{1}H COSY spectrum of **3**.

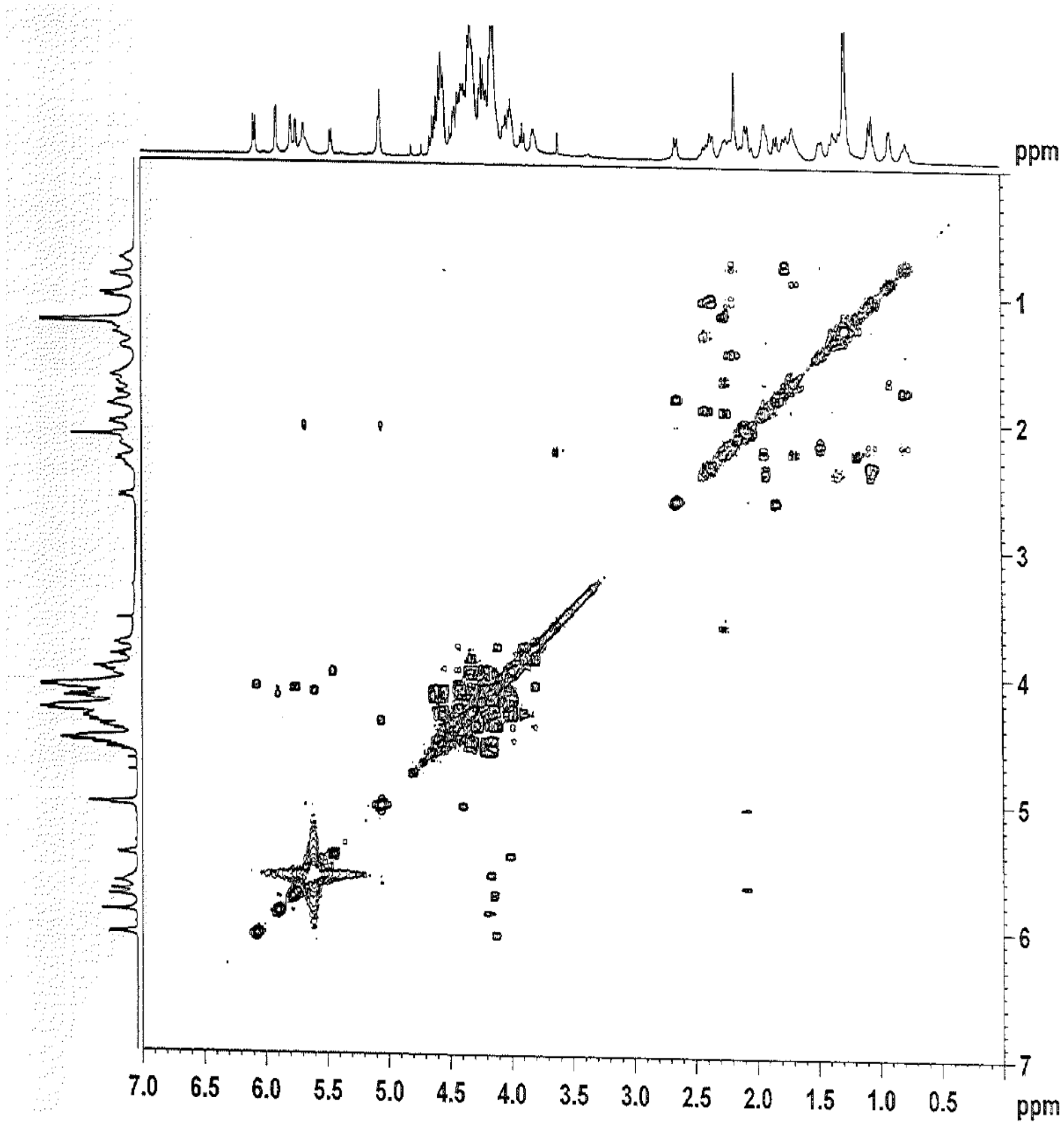

(**c**) HSQC-TOCSY spectrum of **3**.

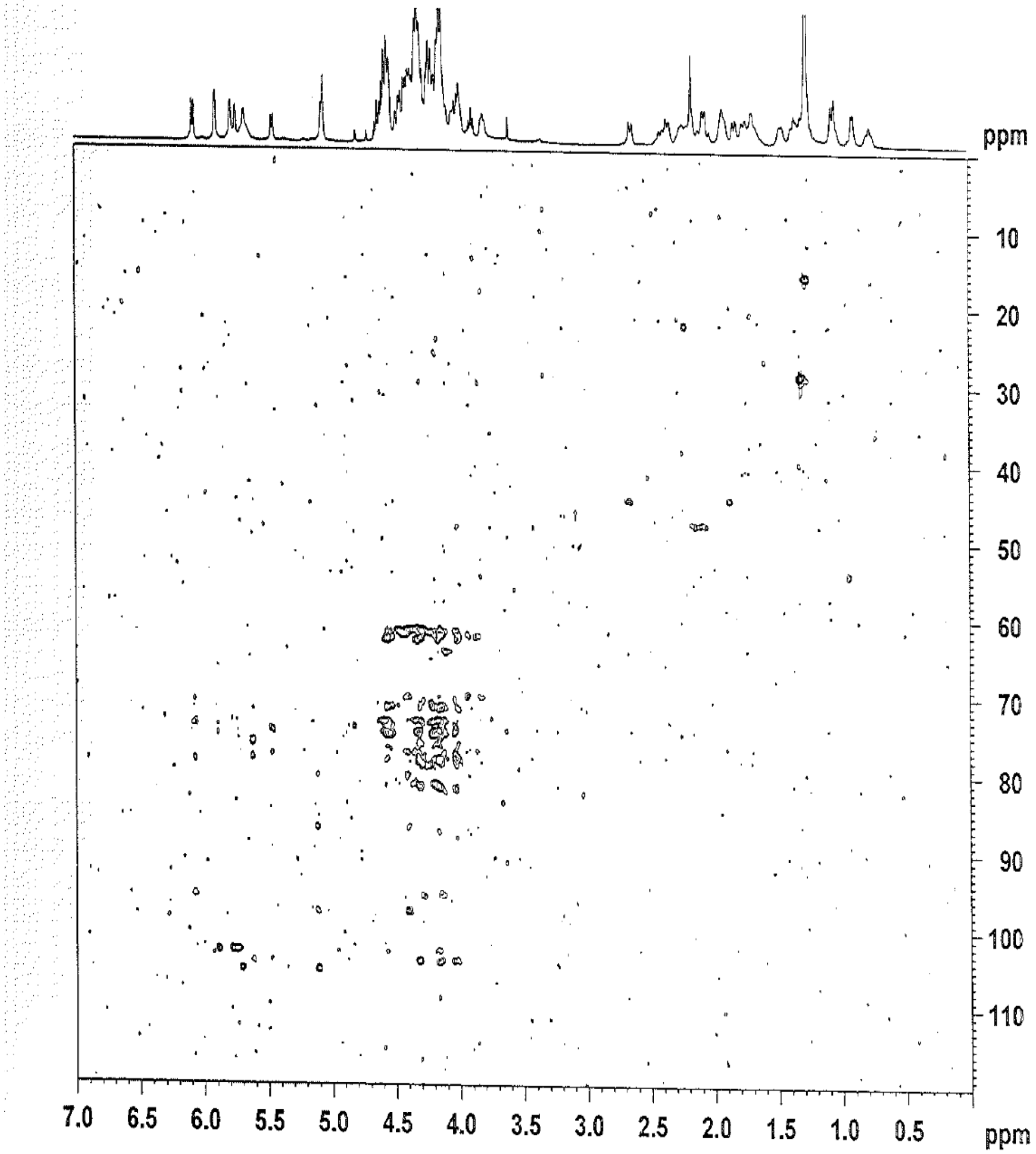

(**d**) HSQC-TOCSY spectrum of **3** (Zoom Version).

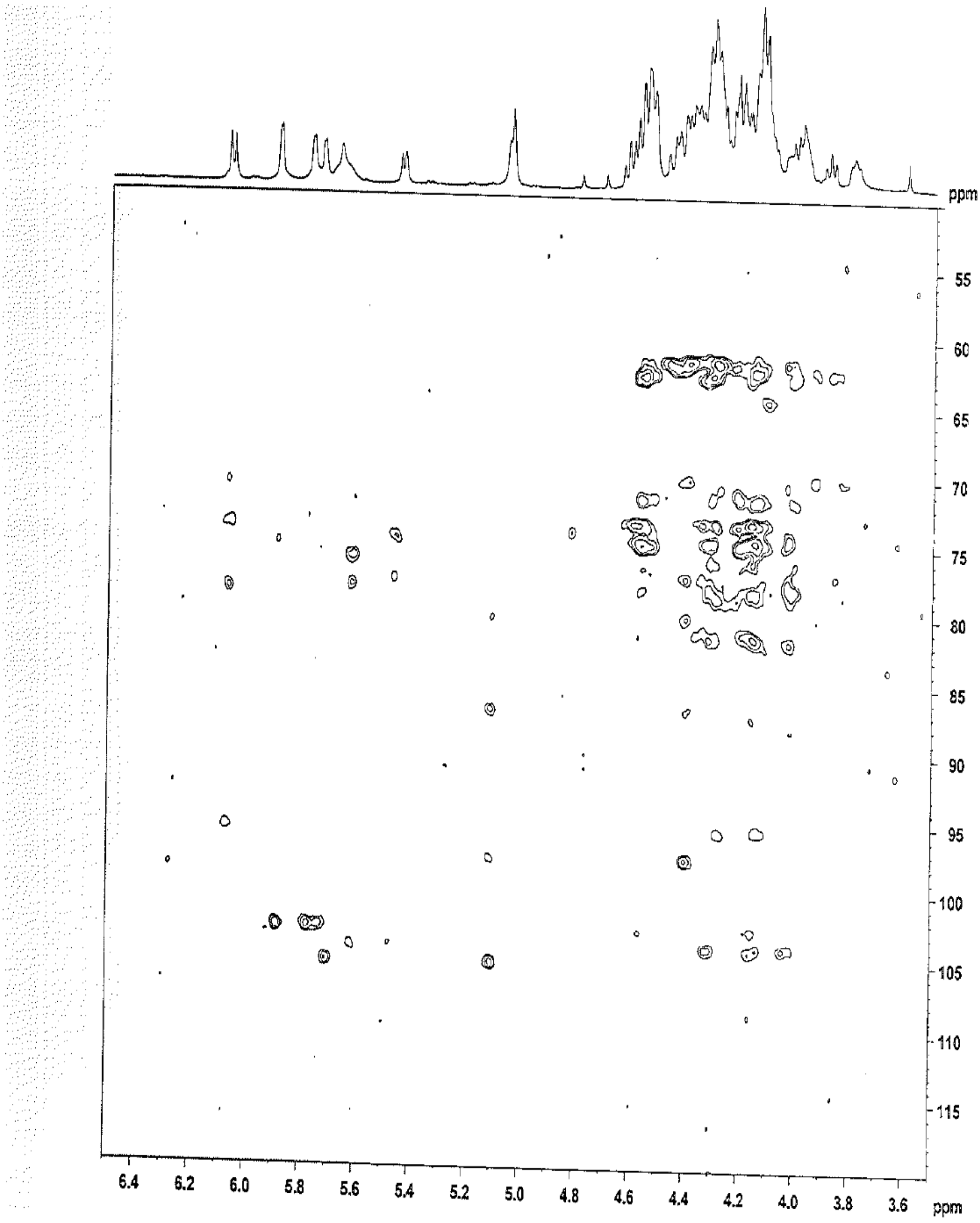

(**e**) ^{1}H-^{13}C HSQC spectrum of **3**.

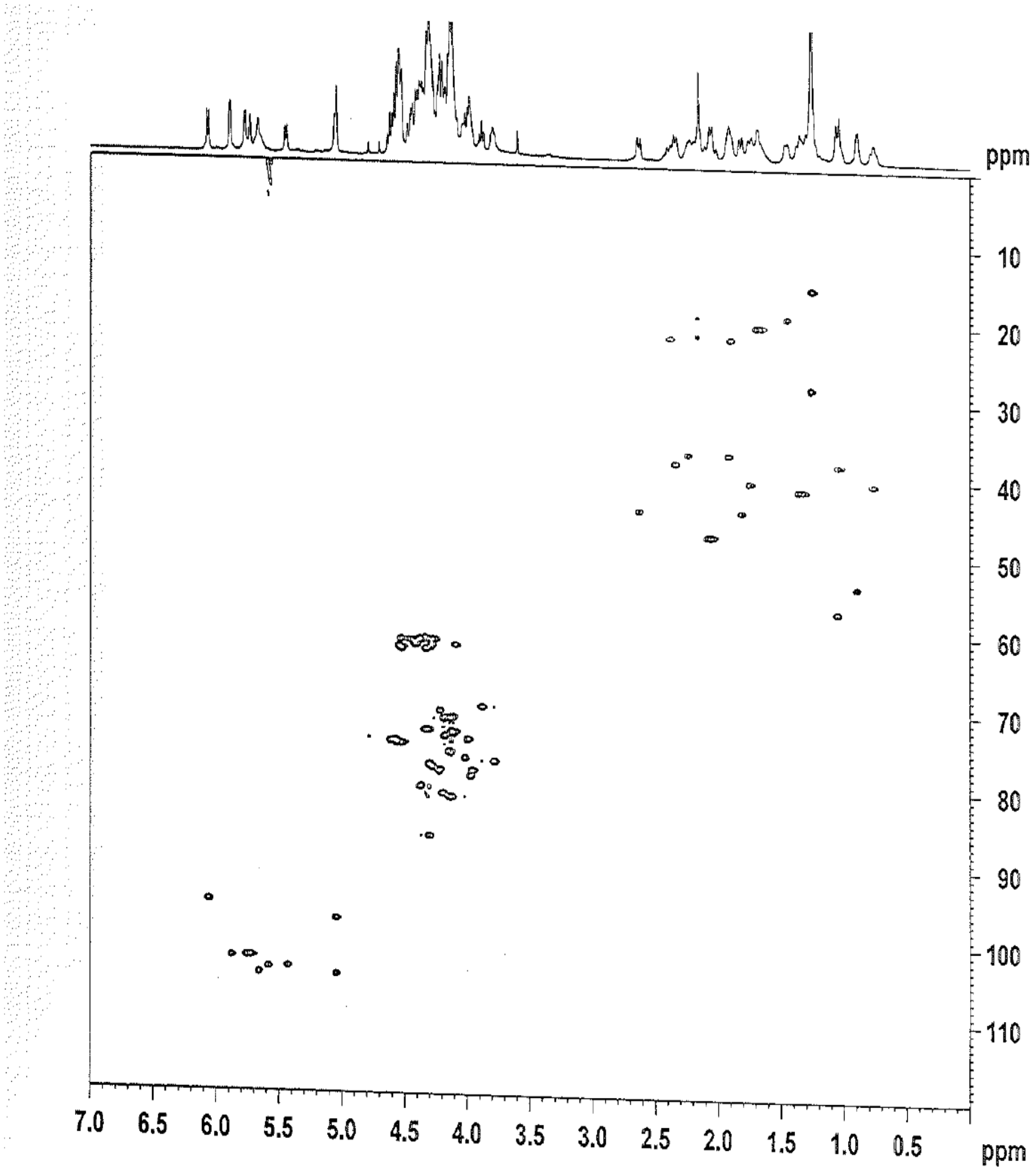

(**f**) ^{1}H-^{13}C HSQC spectrum of **3** (Zoom Version).

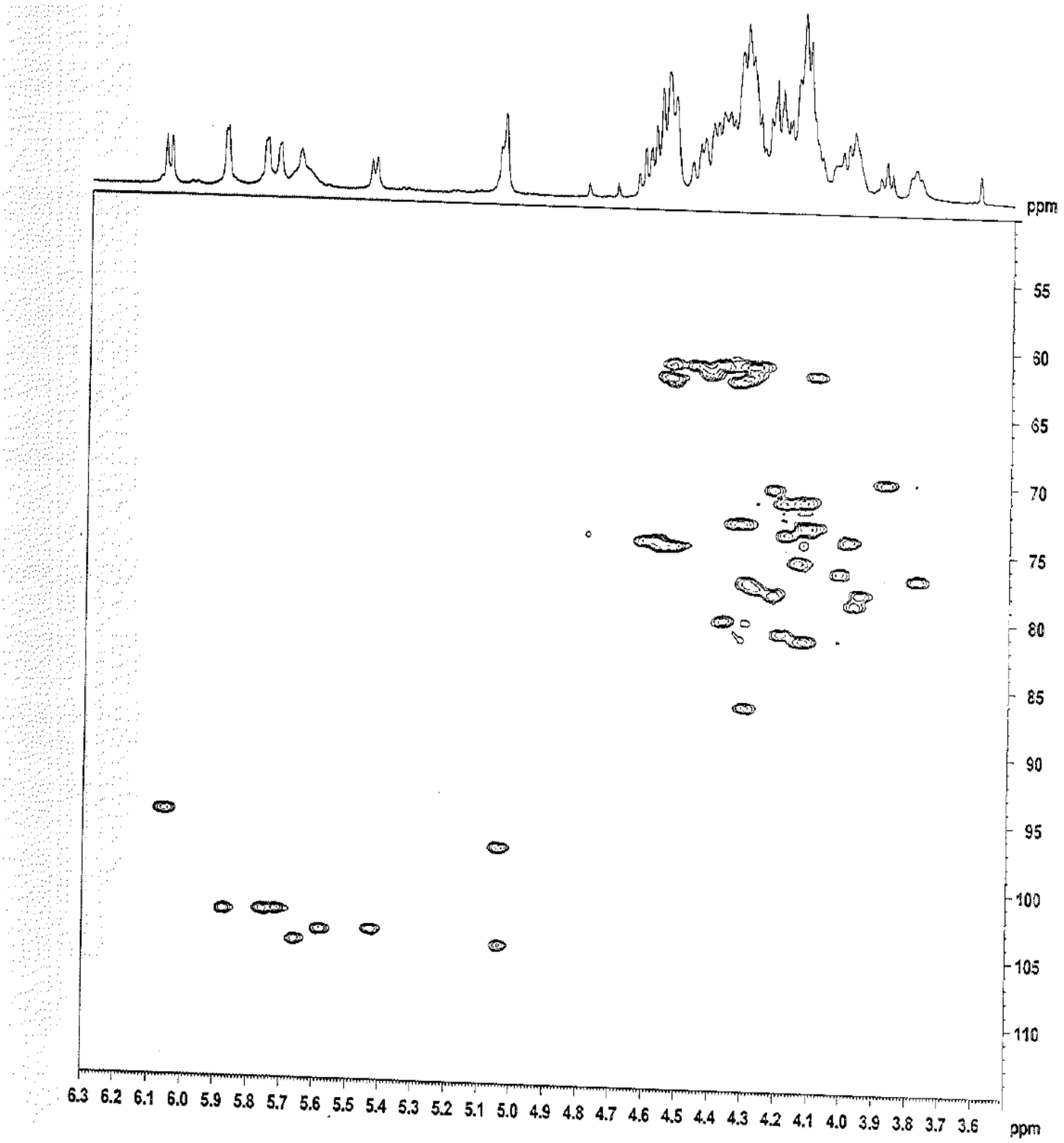

(g) ^{1}H-^{13}C HMBC spectrum of **3**.

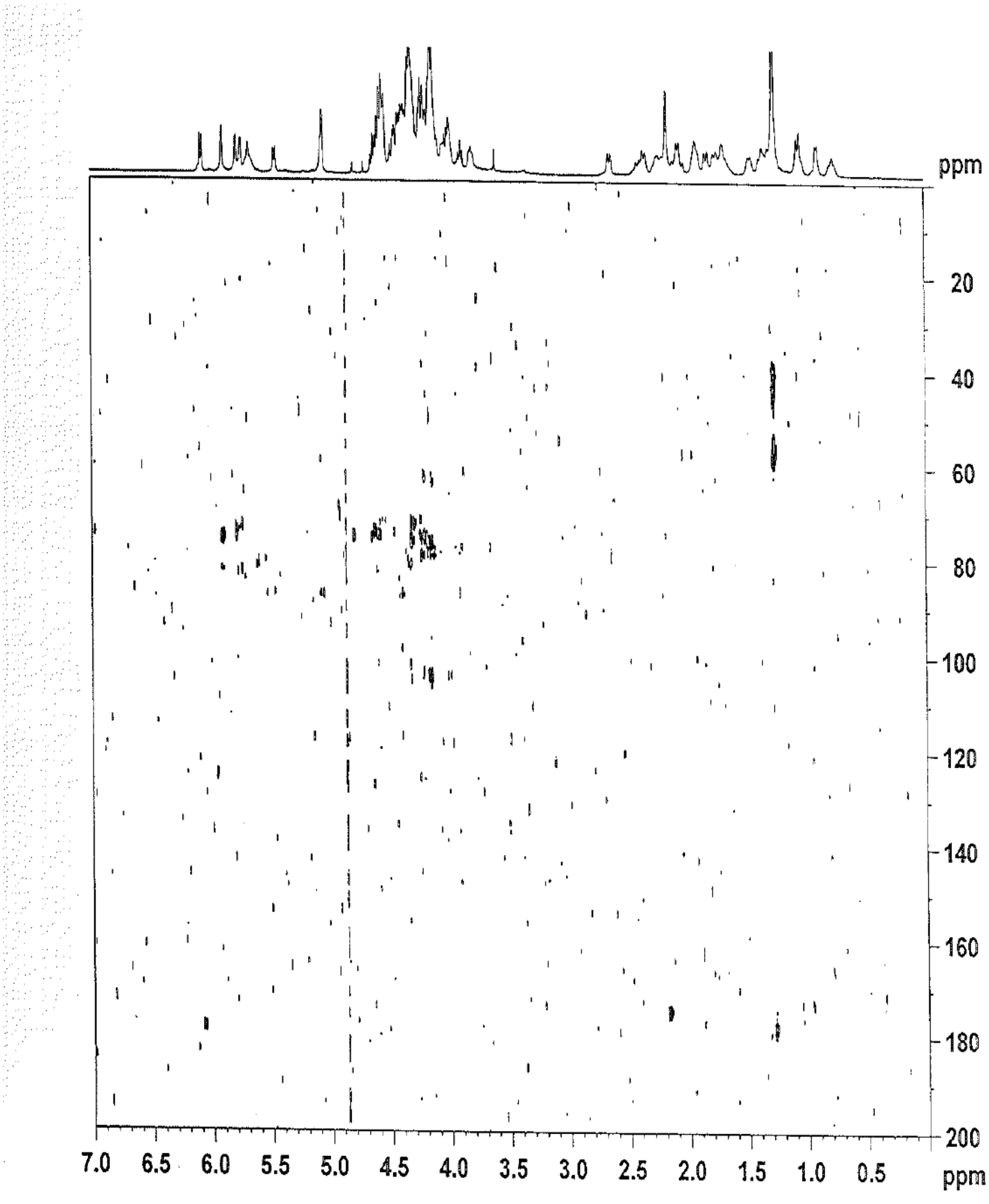

(**h**) ^{1}H-^{13}C HMBC spectrum of **3** (Zoom Version).

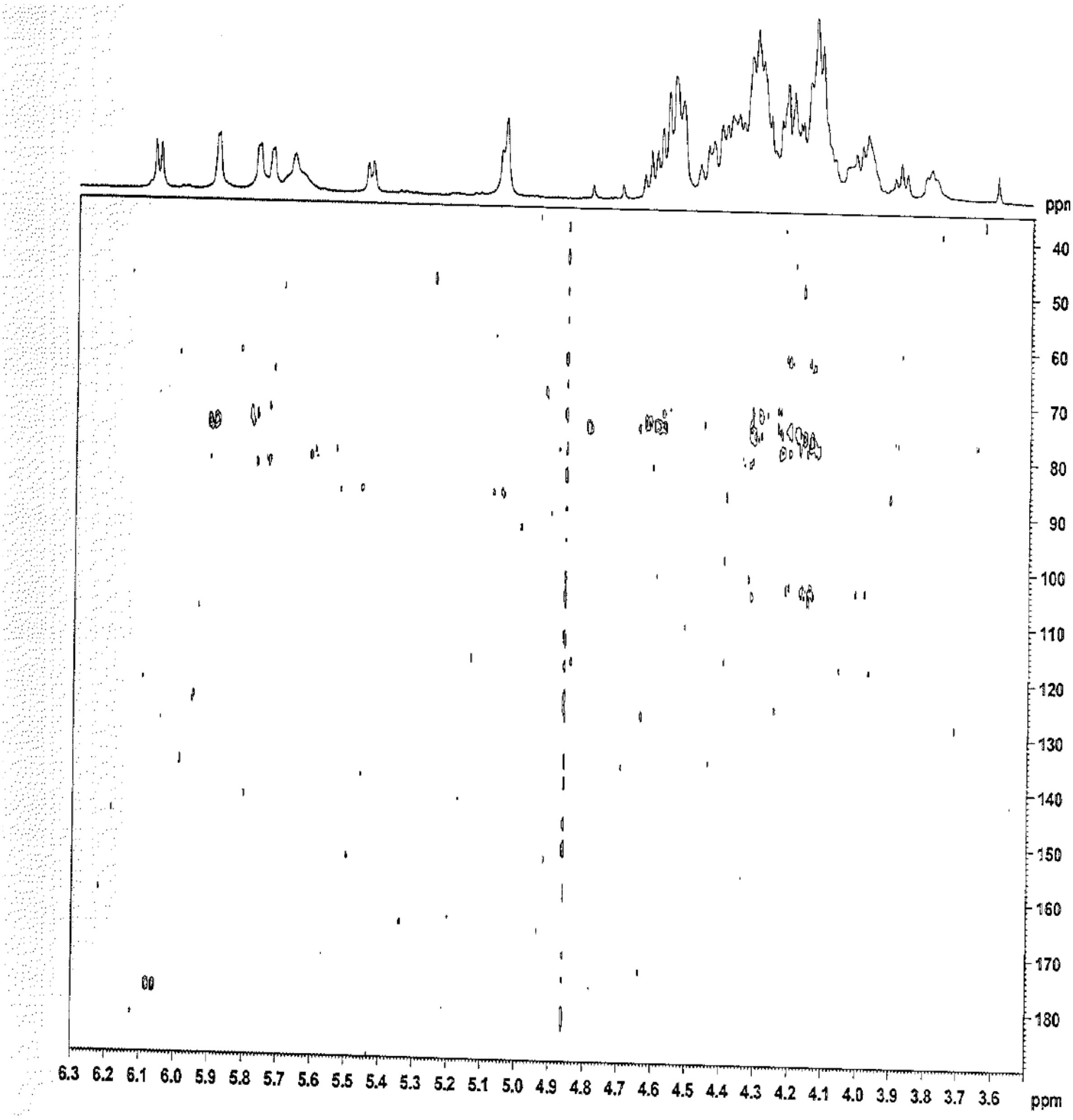

Figure S4. 1D and 2D NMR spectra of 13-[(2-O-β-D-glucopyranosyl-3-*O*-(4-*O*-(4-*O*-(4-*O*-α-D-glucopyranosyl)-α-D-glucopyranosyl)-α-D-glucopyranosyl)-β-D-glucopyranosyl-β-D-glucopyranosyl) oxy] *ent*-kaur-16-en-19-oic acid-[(4-*O*-α-D-glucopyranosyl-β-D-glucopyranosyl) ester] (**4**).

(**a**) ^{1}H-NMR spectrum of **4**.

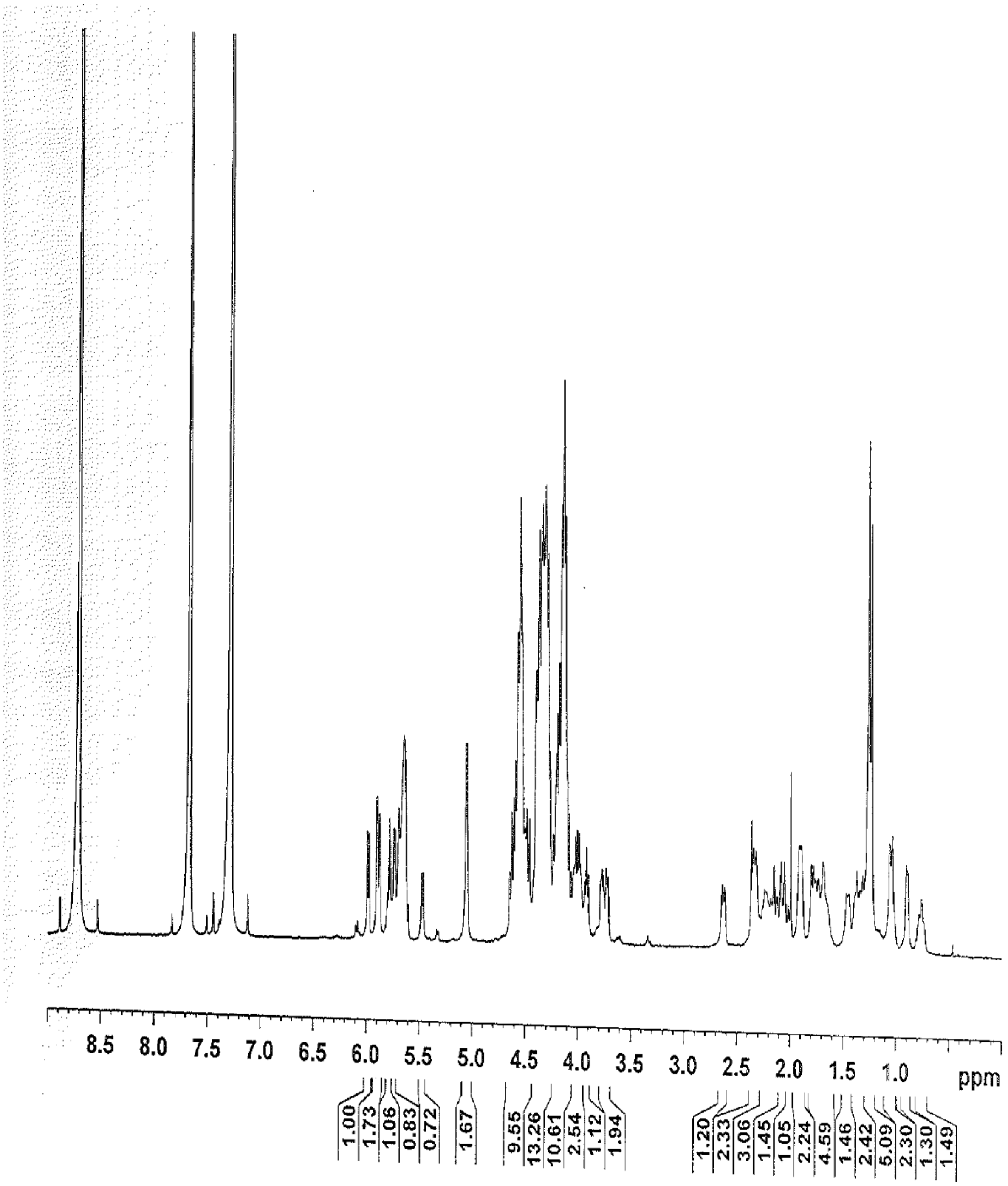

(**b**) ^{1}H-^{1}H COSY spectrum of **1**.

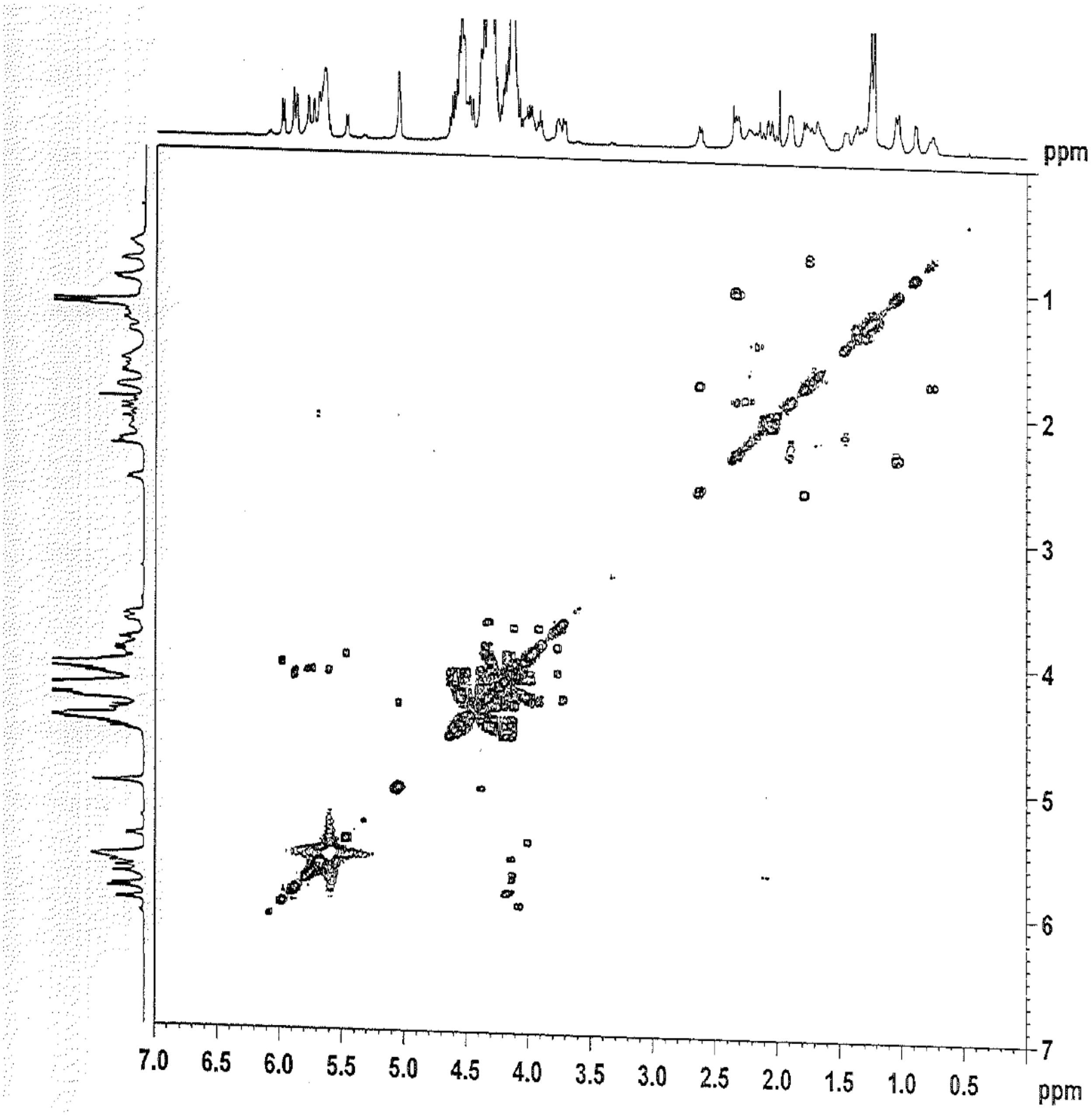

(**c**) ^{1}H-^{13}C HSQC spectrum of **4**.

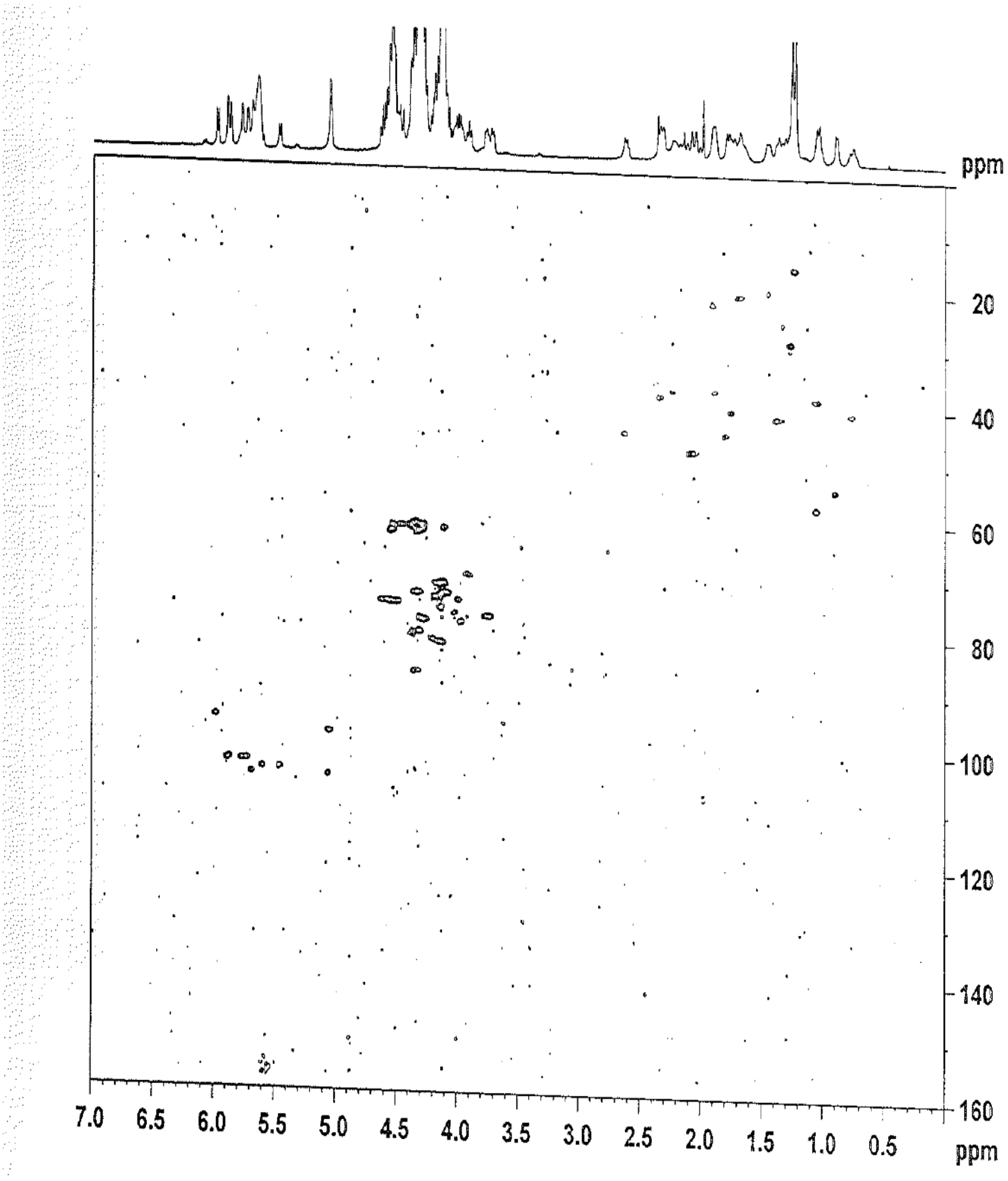

(**d**) ^{1}H-^{13}C HSQC spectrum of **4** (Zoom Version).

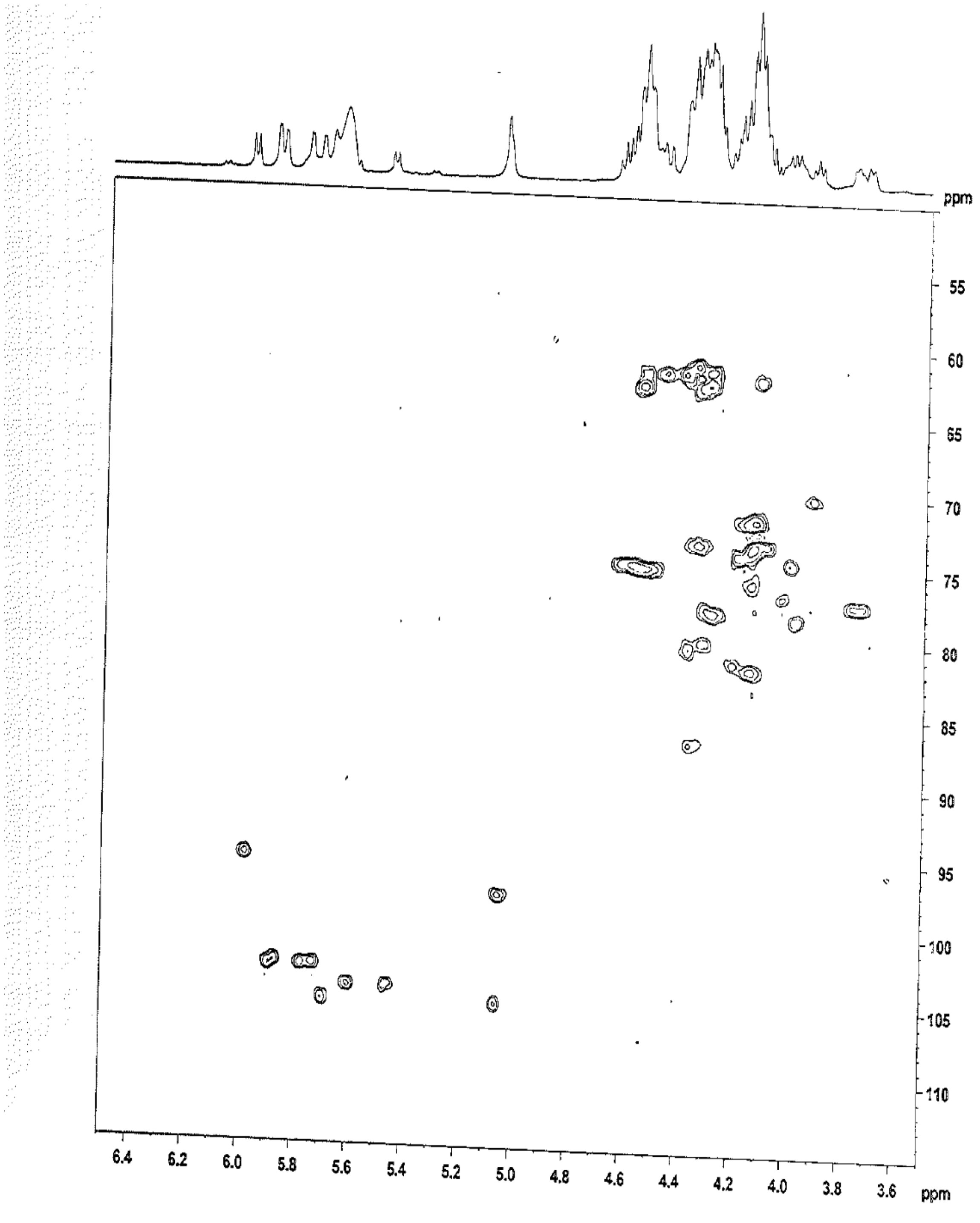

(**e**) ^{1}H-^{13}C HMBC spectrum of **4**.

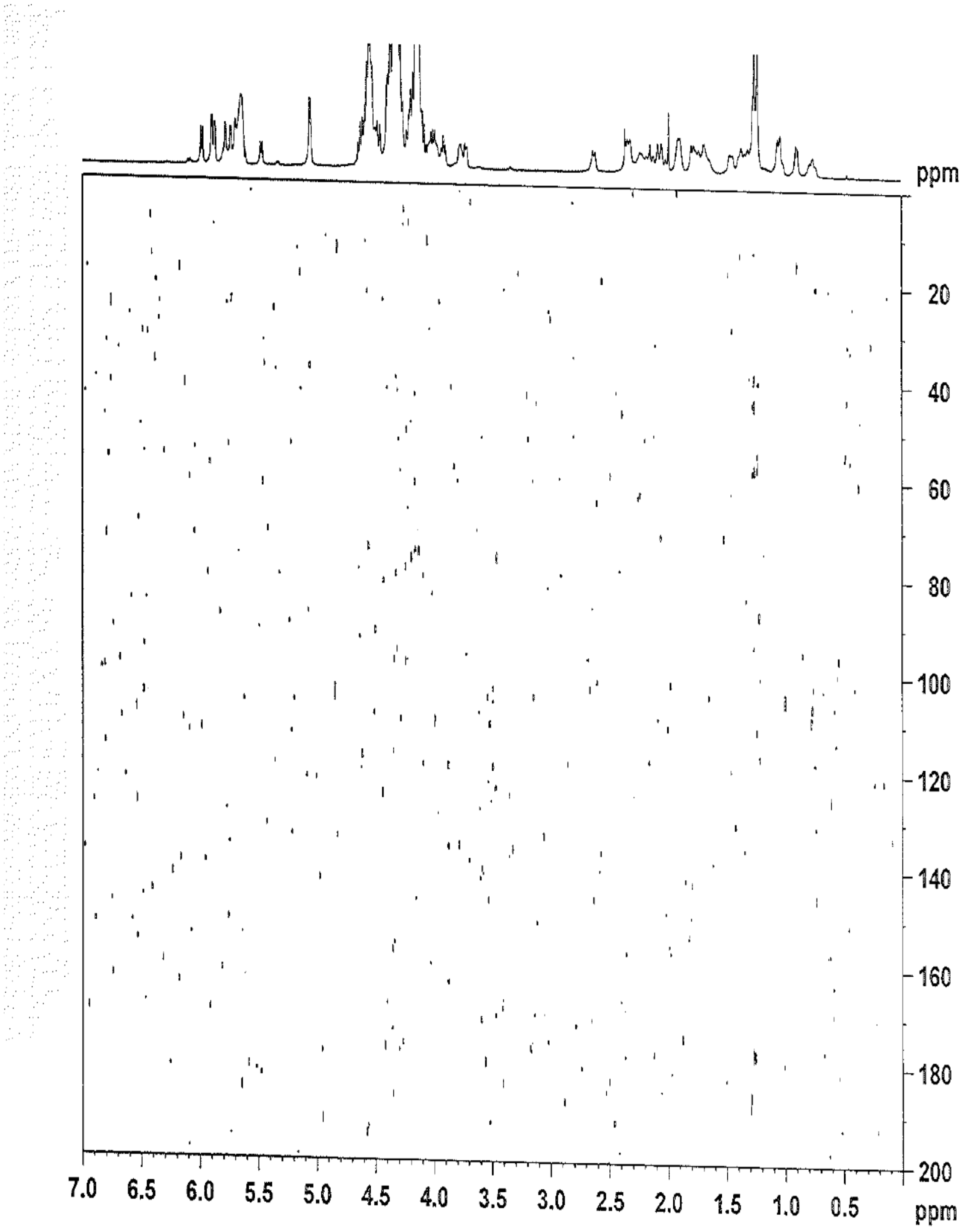

(**f**) ^{1}H-^{13}C HMBC spectrum of **4** (Zoom Version).

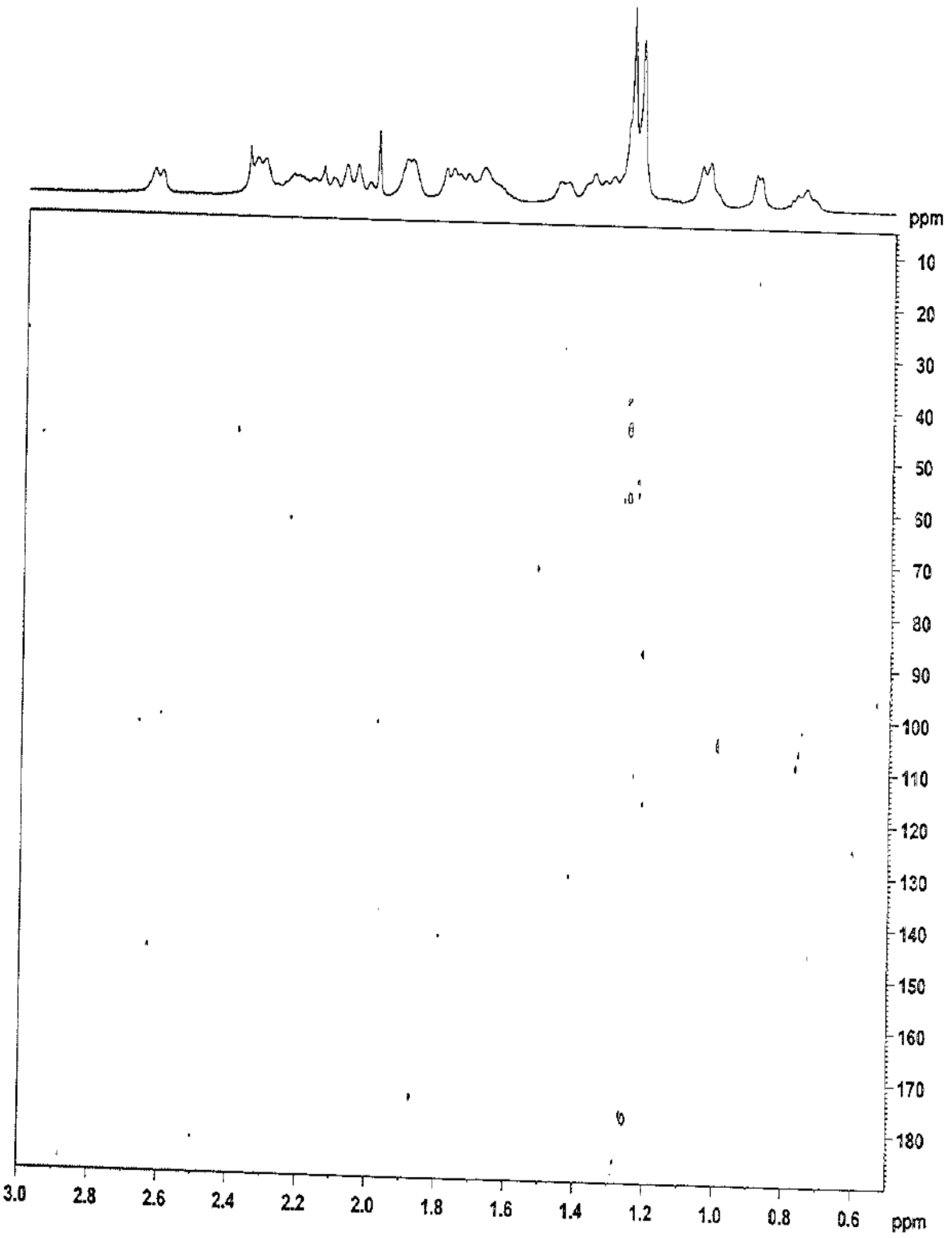

Supplement: Supplementary file 1 [file molecules-19-20280-s001.pdf]
